# Group A streptococcus induces CD1a-autoreactive T cells and promotes psoriatic inflammation

**DOI:** 10.1126/sciimmunol.add9232

**Published:** 2023-06-02

**Authors:** Yi-Ling Chen, Jessica Soo Weei Ng, Rosana Ottakandathil Babu, Jeongmin Woo, Janina Nahler, Clare S. Hardman, Prathiba Kurupati, Lea Nussbaum, Fei Gao, Tao Dong, Kristin Ladell, David A. Price, David A. Duncan, David Johnson, Uzi Gileadi, Hashem Koohy, Graham S. Ogg

**Affiliations:** 1 MRC Human Immunology Unit, MRC Weatherall Institute of Molecular Medicine, University of Oxford, UK; 2 CAMS-Oxford International Centre for Translational Immunology, University of Oxford, UK; 3 Division of Infection and Immunity, School of Medicine, Cardiff University, UK; 4 Systems Immunity Research Institute, School of Medicine, Cardiff University, UK; 5 Diamond Light Source, Harwell Science and Innovation Campus, Didcot, UK; 6 Department of Plastic and Reconstructive Surgery, John Radcliffe Hospital, Oxford University Hospitals National Health Services Foundation Trust, Oxford, UK; 7 Alan Turing Fellow in Health and Medicine, UK

## Abstract

Group A streptococcus (GAS) infection is associated with multiple immunological clinical sequelae, including different subtypes of psoriasis. Whilst such post-streptococcal disorders have been long known, it is a largely unexplained clinical observation. CD1a is expressed at constitutively high levels by Langerhans cells and presents exogenous and endogenous lipid antigens to T cells, but the potential relevance to GAS infection has not been studied. Here we investigated whether GAS-responsive CD1a-restricted T cells contribute to the pathogenesis of psoriasis. We found that healthy individuals have high frequencies of circulating and cutaneous GAS-responsive CD4^+^ and CD8^+^ T cells with rapid effector functions, including production of IL-22. Human skin and blood single-cell CITE-seq analyses of IL-22-producing T cells showed a type 17 signature with proliferative potential, while IFNγ-producing T cells displayed cytotoxic T lymphocyte (CTL) characteristics. Furthermore, individuals with psoriasis had significantly higher frequencies of circulating GAS-reactive T cells, which were enriched for markers of activation, cytolytic potential and tissue association. In addition to responding to GAS, subsets of *in vitro* expanded GAS-reactive T cell clones/lines were found to be auto-reactive, which included recognition of the self-lipid antigen lysophosphatidylcholine. CD8^+^ T cell clones/lines were able to produce cytolytic mediators and lyse infected CD1a-expressing cells. Furthermore, we established cutaneous models of GAS infection in a humanized CD1a transgenic mouse model and identified enhanced and prolonged local and systemic inflammation, with resolution through a psoriasis-like phenotype. In conclusion, these studies link GAS infection to the CD1a pathway and show that GAS infection promotes proliferation and activation of CD1a-autoreactive T cells, with relevance to post-streptococcal disease including the pathogenesis and treatment of psoriasis.

## Introduction

Psoriasis is a common inflammatory skin disease which carries significant morbidity, as well as being associated with joint, intestinal, metabolic and psychological disease (1). It has been long known that group A streptococcus throat infection can promote guttate psoriasis, but the underlying mechanisms have remained largely unexplained (2, 3). Laryngeal *Streptococcus pyogenes* (a group A streptococcus) and other subsets of β-haemolytic streptococcal infections often proceed exacerbation of some forms of psoriasis, including plaque psoriasis (4, 5). Furthermore, tonsillectomy has been found to protect against plaque psoriasis, at least temporarily (6–8). Recurrent streptococcal throat infection is thought to be a form of immunosusceptibility featuring antibody deficiency and impaired T follicular helper cell function, whereby streptococcal infection drives expansion of skin homing lymphocytes (9–11). Psoriasis risk is also linked to individuals carrying the HLA-Cw*0602 allele, and this has been widely assumed to be related to presentation of peptides by HLA-Cw*0602 to T cells in the skin. It is known that HLA-Cw*0602 predisposes individuals to active streptococcal throat infection, and that HLA-Cw6 can engage the inhibitory receptor KIR2DL1 (12, 13). Cw*0602 gene expression shows relative insensitivity to TNFα− and IFNγ-mediated induction (14, 15), and an enhancer element of HLA-Cw*0602 is thought to explain the early onset of plaque psoriatic disease association (14). HLA-Cw*0602 positive individuals also benefit the most from the protective effect of tonsillectomy (7). Collectively, these findings are compatible with the hypothesis that impaired control of pharyngeal streptococcal infection drives a downstream non-MHC-dependent cutaneous inflammatory response. The underlying pathways remain to be determined.

A number of mechanisms have been proposed to explain the link between GAS and psoriatic disease. T cell cross-reactivity between streptococcal M protein and skin keratins (e.g. keratin 17) has been described, but specific T cells are not present in psoriatic skin lesions (16–19). Furthermore, keratin 17 can be expressed at multiple different epithelia beyond the skin, including proximal intestine, lung and urogenital tract, which are not clinically involved during guttate psoriasis. Streptococcal superantigens have also been implicated in the pathogenesis of psoriasis, but the spatio-temporal relationship between streptococcal throat infection and guttate psoriasis is not fully compatible with such a mechanism; for example, guttate psoriasis typically arises 1-3 weeks after onset of the throat infection rather than at the peak of throat symptoms (3, 20). Oligoclonal αβ T cell expansions have been described in lesional and resolved psoriatic skin, thought to be more in keeping with antigen-driven reactivity than broad superantigen effects (21). T cell responses to LL-37 and melanocyte ADAMTSL5 have also been described, but have not yet been linked to streptococcal infection in the setting of psoriasis (22, 23). Overall, while these data confirm that a streptococcal-induced immune response can promote psoriatic inflammation, the specific pathways are yet to be fully explained.

CD1a is a relatively non-polymorphic HLA class I-like molecule expressed at constitutively high levels by Langerhans cells (24). It can also be expressed by thymocytes and induced on dendritic cell subsets, T cells and innate lymphoid cells (25, 26). CD1a presents endogenous and exogenous lipid antigens to T cells, inducing pro-inflammatory cytokines with relevance to psoriasis, including IL-22, IL-17A and IFNγ (27–31). Elevated frequencies of CD1a-reactive T cells have been found in the blood and skin of patients with psoriasis, and imiquimod-induced inflammation is associated with exacerbated disease in a human CD1a transgenic mouse model (29, 32). T cell recognition of permissive skin lipid antigens can be mediated, at least in some cases, through TCR engagement of the A’ roof of CD1a without direct lipid:TCR contact, helping to explain broad lipid reactivity (33, 34). CD1a-reactive T cells have been shown to respond to mycobacterial and staphylococcal antigens, implicating a role in bacterial defence (24, 28, 35, 36). However, there are no studies that have investigated the role of CD1a reactivity in the immune response to Group A streptococcus, and the consequences for associated inflammatory disease. Here, we test the hypothesis that Group A streptococcus induces CD1a reactivity, and investigate the underlying mechanisms and relevance to psoriasis, with therapeutic implications.

## Results

### Healthy individuals have high frequencies of circulating and cutaneous GAS-responsive CD1a-reactive T cells

**Figure 1:**
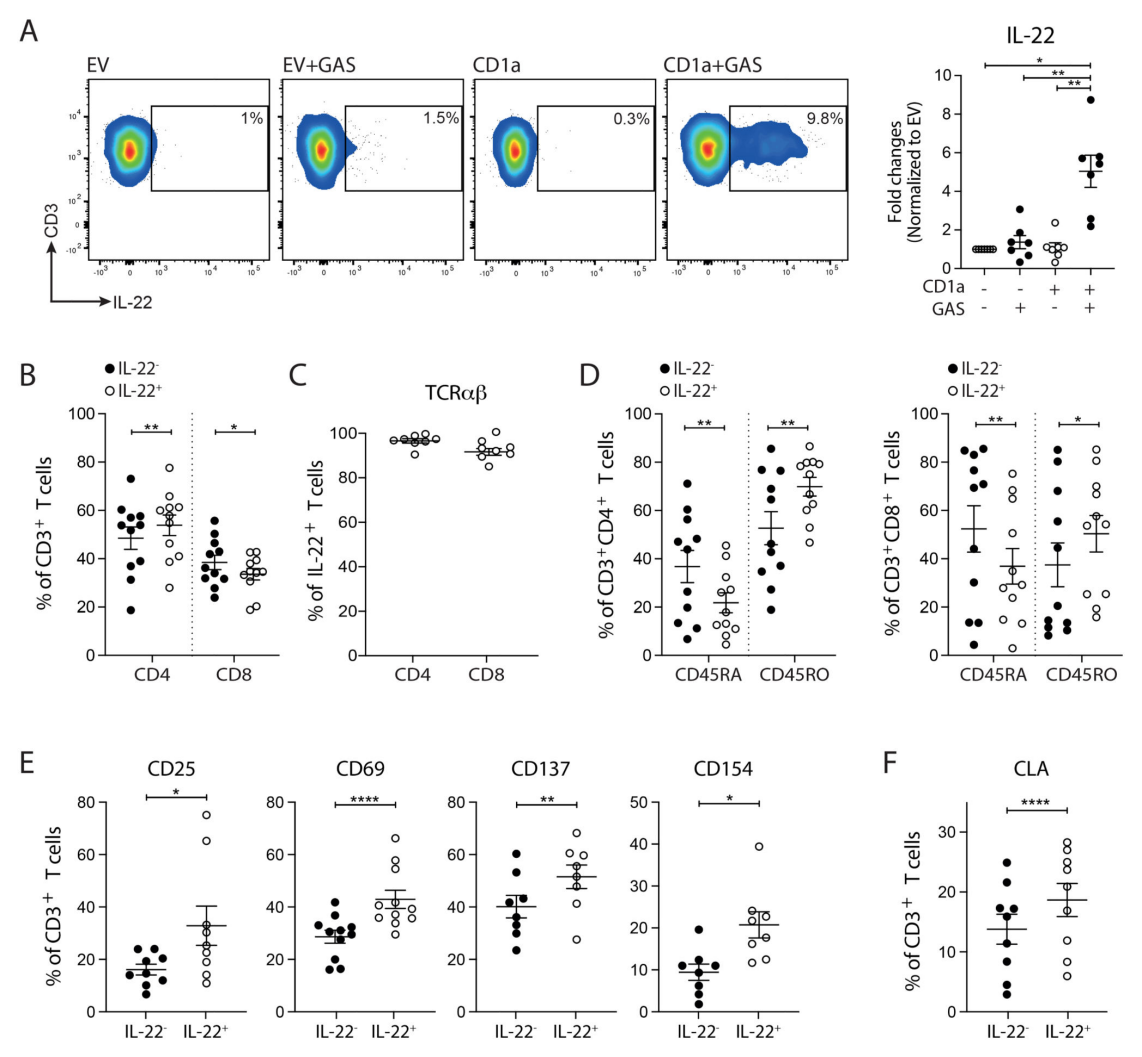
High frequencies of circulating GAS-responsive CD1a-reactive T cells found in healthy individuals. **(A)** Production of IL-22 from polyclonal blood T cells after 4-hour co-culture with control or GAS-infected K562 cells (MOI=100) detected by Secretion assay. One representative result is shown. Percentages of **(B)** CD4^+^, CD8^+^ and **(C)** TCRαβ^+^ population in IL-22-secreting GAS-responsive T cells analyzed by flow cytometry. **(D)** Percentages of CD45RA^+^ and CD45RO^+^ in IL-22-secreting GAS-responsive CD4^+^ and CD8^+^ T cells analyzed by flow cytometry. Expression of **(E)** CD25, CD69, CD137, CD154 and **(F)** CLA on IL-22-secreting GAS-responsive T cells analyzed by flow cytometry. Each symbol represents an individual donor (mean ± SEM) (n=7-11). *P < 0.05, **P < 0.01 and ****P < 0.0001; repeated-measures (RM) one-way ANOVA with Tukey's post hoc test (A, B) or two-tailed paired t test (D, E, F). Data are representative of more than three independent experiments.

To determine whether GAS-responsive CD1a-reactive T cells were present across a healthy cohort, we utilized CD1a-transfected K562 cells as target cells. K562 lack HLA class I and II, and mimic CD1a antigen presentation by primary antigen-presenting cells (29, 37, 38). K562-CD1a cells infected with GAS were recognized by *ex vivo* polyclonal T cells in a CD1a-dependent manner, leading to production of IL-22 (Figure 1A, left panel). All healthy adults tested had detectable GAS-responsive CD1a-reactive T cells, comprising a large population of circulating T cells (Figure 1A, right panel). High frequencies of CD1a-reactive T cells have been predicted from *in vitro* expansions, but not yet proven in *ex vivo* analyses (31, 39). The use of GAS as an antigen driver thus allowed the demonstration that CD1a-reactive T cells represent a large population of the circulating T cell repertoire and prompted our continued investigation of the nature of the T cell response.

Through gating on the IL-22-producing GAS-responsive T cells, we identified that this population comprised both CD4^+^ and CD8^+^ T cells, with slight enrichment of the CD4^+^ populations (Figure 1B). They predominately expressed αβ TCRs (Figure 1C) and were enriched for CD45RO expression, consistent with previous antigen exposure (Figure 1D) and existing findings (31, 39). As expected, the IL-22-producing GAS-responsive T cells were enriched for markers of T cell activation (Figure 1E). These cells also had elevated expression of the skin homing marker, cutaneous lymphocyte associated antigen (CLA), implicating a requirement for peripheral control of T cells that have the potential capacity to home to the skin (Figure 1F).

**Figure 2:**
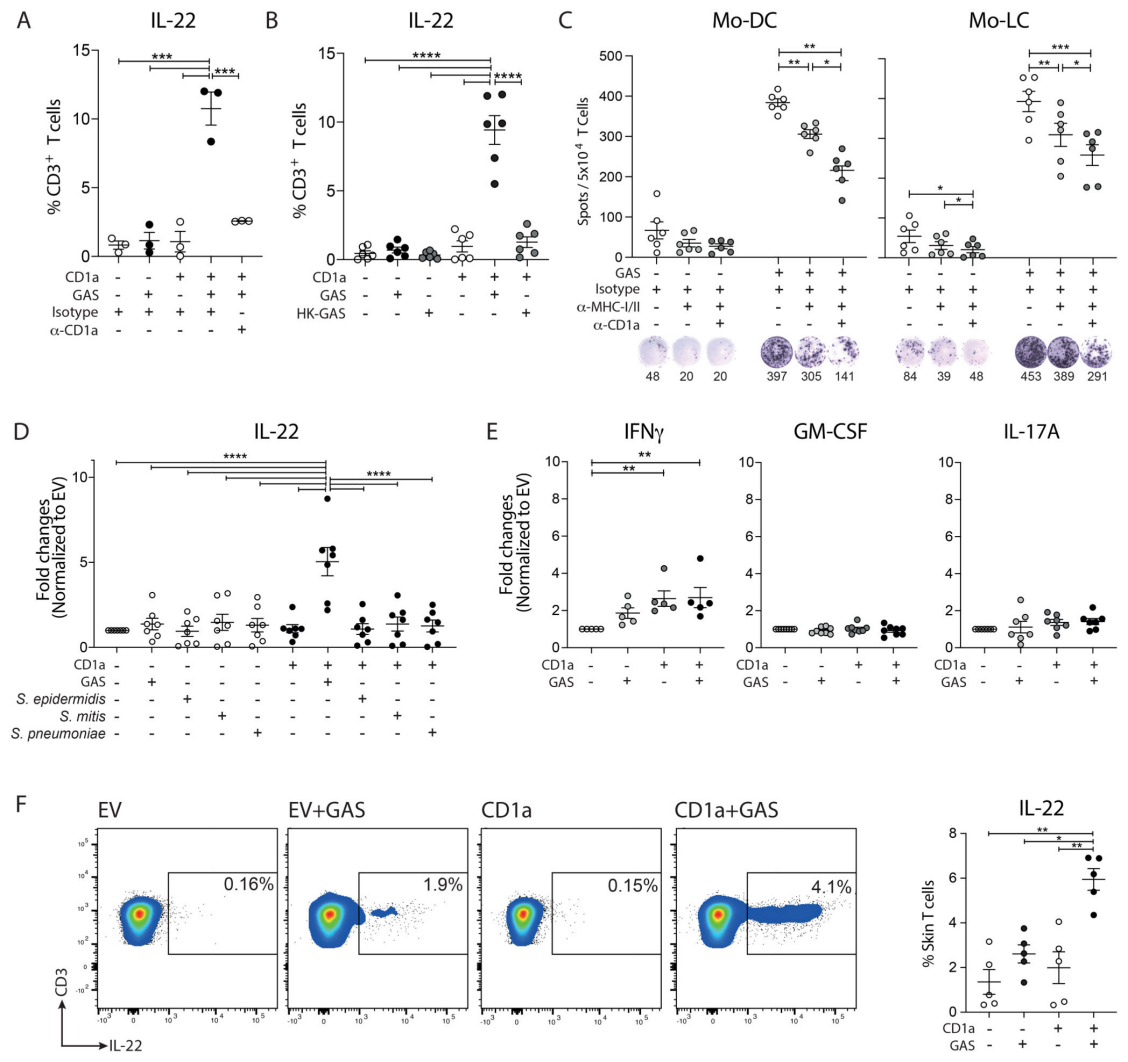
High frequencies of cutaneous GAS-responsive CD1a-reactive T cells found in healthy individuals. **(A)** Production of IL-22 from polyclonal blood T cells detected by Secretion assay after 4-hour co-culture with control and GAS-infected K562 cells (MOI=100) in the presence of anti-CD1a or control IgG (10 µg/ml) (n=3). **(B)** Production of IL-22 from polyclonal blood T cells detected by Secretion assay after 4-hour co-culture with heat-inactivated GAS-infected K562 cells (n=6). **(C)** Secretion of IL-22 from autologous blood T cells assessed by ELISpot after 16-hour co-culture with control or GAS-infected mo-DCs or LC-like cells (MOI=20) in the presence of anti-CD1a or control IgG (10 µg/ml). Anti-HLA-A,B,C (10 µg/ml) and HLA-DR (10 µg/ml) were added to block peptide-specific T cell response. One representative result is shown of three independent experiments (n=6). **(D)** Production of IL-22 from polyclonal blood T cells detected by Secretion assay after 4-hour co-culture with control, GAS-, *S. epidermidis*-, *S. mitis*-, and *S. pneumoniae*-infected K562 cells (MOI=50) (n=7). **(E)** Production of IFNγ, GM-CSF, and IL-17A from polyclonal T cells detected by Secretion assay after 4-hour co-culture with control or GAS-infected K562 cells (MOI=50) (n=5-8). **(F)** Production of IL-22 from polyclonal skin T cells after 4-hour co-culture with control or GAS-infected K562 cells (MOI=50) detected by Secretion assay. One representative result is shown (n=5). Each symbol represents an individual donor (mean ± SEM). *P < 0.05, **P < 0.01, ***P < 0.001 and ****P < 0.0001; two-way ANOVA with Tukey's post hoc test (A, B, C, D), and repeated-measures (RM) one-way ANOVA with Tukey's post hoc test (E, F). Data are representative of three or more independent experiments.

Having identified a population of GAS-responsive T cells in healthy individuals, we went on to investigate their CD1a-dependence, and to test whether primary CD1a-expressing target cells could also mediate antigen presentation. Anti-CD1a blockade was able to effectively inhibit recognition of GAS-infected K562-CD1a cells by IL-22-producing polyclonal *ex vivo* blood T cells (Figure 2A), suggesting the possibility of therapeutic intervention in GAS-driven inflammatory skin disease. Heat-killed GAS was not able to induce a T cell response (Figure 2B), suggesting a requirement for active K562-CD1a infection. These findings also rule out a role for heat-sensitive soluble mediators such as some TLR ligands. We next showed that both autologous monocyte-derived dendritic cells (mo-DCs) and Langerhans cell-like cells (LC-like DCs) were able to present GAS-associated antigens to polyclonal T cells in a CD1a-dependent manner (Figure 2C). Of note, the blockade of MHC class I/II and CD1a was additive, suggesting the pathways are acting in parallel to present peptide antigens as well as CD1a-dependent lipid-driven responses (26, 33, 38, 39). These data confirmed that *ex vivo* polyclonal IL-22-producing T cells were able to respond to primary CD1a-expressing cells infected with GAS. Only GAS was capable of inducing CD1a-reactivity among the streptococcal and staphylococcal species tested (Figure 2D). In addition, we were able to observe IFNγ-producing CD1a-autoreactive T cell responses from healthy individuals, but no further net increase was observed after GAS infection (Figure 2E). Furthermore, limited GM-CSF or IL-17A-producing T cells were detected (Figure 2E). These observations were further investigated in subsequent experiments. We next investigated whether GAS-responsive CD1a-reactive T cells were present in healthy skin and found high frequencies of IL-22-producing cells in all individuals tested (Figure 2F).

Overall, the existence of a high frequency of GAS-responsive CD1a-reactive T cells with rapid effector function is compatible with a requirement for these cells in defence against a ubiquitous and potentially lethal pathogen.

### IL-22- and IFNγ-secreting CD1a-reactive GAS-responsive T cells exhibit diverse functionalities

**Figure 3:**
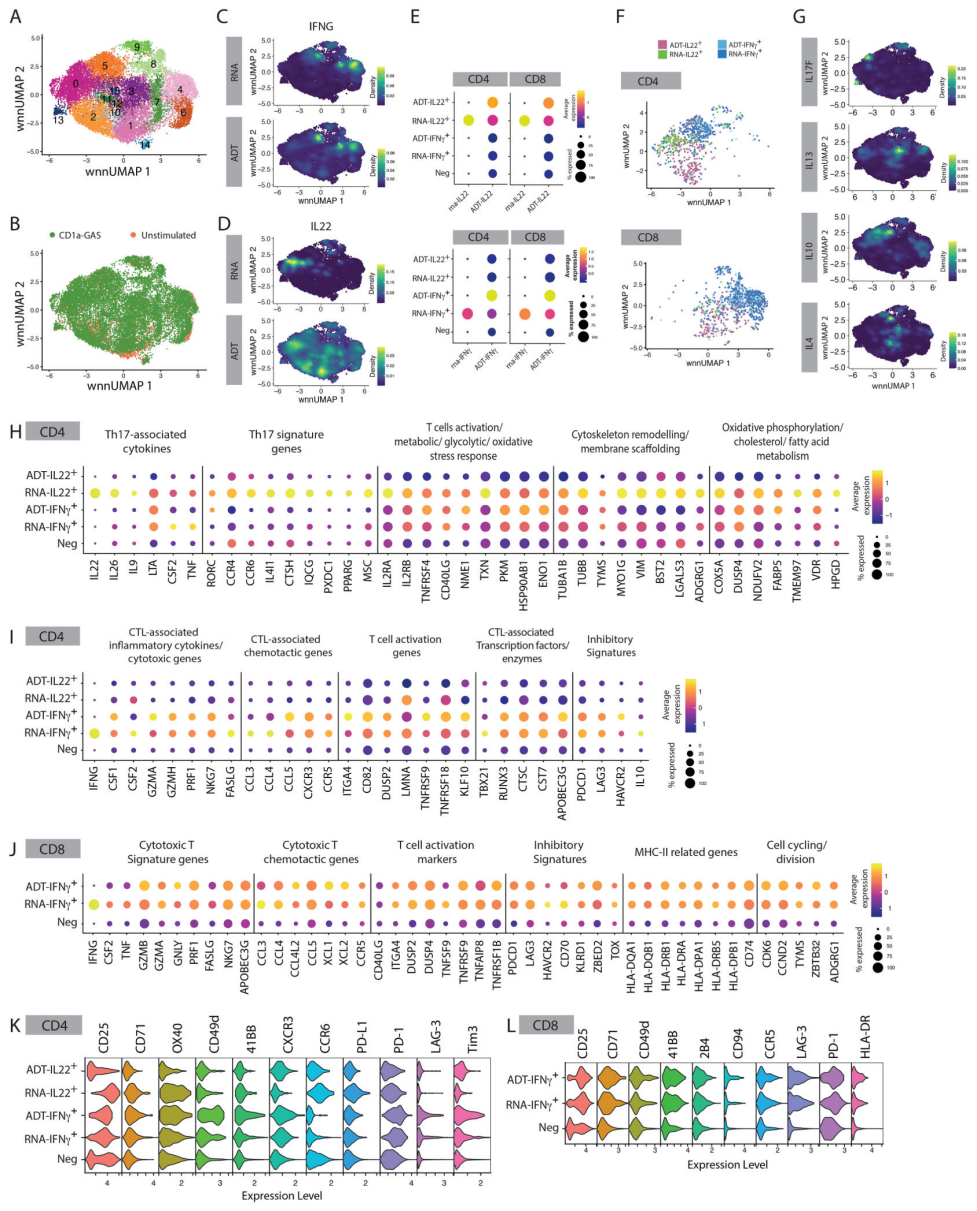
scRNA-seq reveals diverse functionalities of the IL-22- and IFNγ-secreting CD1a-reactive GAS-responsive T cells. Single-cell multi-omic analysis of skin CD3^+^ cells after 6-hour co-culture with GAS-infected K562-CD1a cells (MOI=50) **(A)** UMAP plot showing unbiased clustering of the skin CD3^+^ cells. **(B)** UMAP plot with cell clusters identified based on the co-culture conditions (GAS-infected K562-CD1a (n=4) vs. unstimulated control (n=2)). Nebulosa plots showing mRNA and protein expression density of IFNγ **(C)** and IL-22 **(D)** from skin CD3^+^ cells. **(E)** Dot plots showing the gene expression signatures of IL-22- or IFNγmRNA and protein (ADT) level of the ADT-IL-22^+^, RNA-IL-22^+^, ADT-IFNγ^+^, RNA-IFNγ^+^ and IL22^-^IFNγ^-^ (Neg) skin T cells. **(F)** UMAP plot showing the clustering relation of the CD4^+^ and CD8^+^ ADT-IL-22^+^, RNA-IL-22^+^, ADT-IFNγ^+^, RNA-IFNγ^+^ skin T cells. **(G)** Nebulosa plots showing gene expression density of *IL-17F, IL-13, IL-10*, and *IL-4* from skin CD3^+^ cells. Dot plots showing the gene expression signatures of IL-22-producing skin CD4^+^ T cells **(H)**, IFNγ-producing skin CD4^+^ T cells **(I)**, and IFNγ-producing skin CD8^+^ T cells **(J)**. Violin plots showing the surface marker expressions (ADT) signatures of IL-22- and IFNγ-producing skin CD4^+^ T cells **(K)**, and IFNγ-producing skin CD8^+^ T cells **(L)**.

We next used single-cell analyses to test whether the T cells were enriched for particular subsets and whether they showed features of functional relevance, such as skin residence and activatory/inhibitory receptor expression. A cellular indexing of transcriptomes and epitopes by sequencing (CITE-seq) dataset comprising GAS-responsive CD1a-reactive skin T cells was constructed using our previous K562-CD1a stimulation strategy to FACS isolate IL-22- or IFNγ-producing T cells. Non-IL-22/IFNγ-producing and *ex vivo* unstimulated skin T cells were included to establish phenotypic baseline. We identified 15 phenotypically distinct clusters (Figure 3A), with each cluster comprising cells from each donor (Figure S1A-B). A degree of spatial separation was observed of T cells derived from each treatment condition (Figure 3B, Figure S1C), and of CD4-expressing T helper cells (Th) and CD8-expressing T cytotoxic cells (Tc) (Figure S1D). Protein CD45RO, CD25, CD11a and CD69 expression were used to confirm that skin contains predominantly antigen-experienced T cell subsets (Figure S1E-F).

CITE-seq antibodies against the fluorochromes PE or APC on the detection antibodies were included to further characterize IL-22- or IFNγ-producing skin T cells, respectively. We observed a good association between mRNA and protein expression for IFNγ (Figure 3C), but IL-22 protein was expressed by more cells than *IL22* RNA (Figure 3D). Such discordancy is well-described and may reflect protein/RNA analytical timing and emphasizes the importance of such RNA/protein multimodal analyses (40). Therefore, to capture all relevant populations, we grouped the CD1a-responding cells into five subgroups based on their mRNA and protein (ADT) expression of IL-22 or IFNγ: ADT-IL-22^+^, RNA-IL-22^+^, ADT-IFNγ^+^, RNA-IFNγ^+^, and Neg (IL-22^-^IFNγ^-^) (Figure 3E). ADT-IL-22^+^ and RNA-IL-22^+^ T cells were enriched in clusters 1/2 and cluster 0, respectively, while ADT-IFNγ^+^ and RNA-IFNγ^+^ T cells were concentrated in clusters 4, 5, 8 and 9 (Figure 3F, Figure S1G). In addition to IL-22 and IFNγ, a small proportion of skin T cells could produce IL-17F, IL-10, IL-13 or IL-4 (Figure 3G), indicating a broad spectrum of immune modulatory functions of CD1a-restricted T cells.

We found 480 DEGs and 501 DEGs that characterized each CD4^+^ and CD8^+^ subgroup, respectively (Figure S1H). *IL-22*-expressing CD4^+^ T cells also expressed Th17-associated cytokines (*IL26, IL9*, and *LTA* (Lymphotoxin-α), *CSF2* (GM-CSF) and *TNF* (TNFα)), core Th17 signature genes (*RORC, CCR4, CCR6, IL4LI1, CTSH, IQCG, PXDC1, PPARG, MSC*) (41–44), as well as T cell activation related genes (*IL2RA, IL2RB, TNFRSF4* (OX40), *ITGA4, CD40LG, NME1*), metabolic, glycolytic, and oxidative stress response transcripts (*TXN, PKM, HSP90AB1, ENO1*) (Figure 3H). For example, Thioredoxin (TRX), a small redox protein encoded by *TXN*, is induced by oxidative stresses to protect immune cells from apoptosis, and promotes Th1 differentiation and IFN-γ production in T cells (45–47). Enolase 1, encoded by *ENO1*, is a critical regulator of the glycolytic and effector activity of CD8+ tumour-infiltrating lymphocytes (48). These cells also showed proliferation and cell adhesion capacity with increased expression of microtubules and cytoskeleton remodelling genes (*TUBA1B, TUBB, TYMS, MYO1G*), membrane scaffolding and organization genes (*VIM, BST2, LGALS3, ADGRG1*), and genes involved in oxidative phosphorylation, cholesterol and fatty acid metabolism (*COX5A, DUSP4, NDUFV2, FABP5, TMEM97, VDR, HPGD*) (Figure 3H). Interestingly *ENO1, VDR* (vitamin D receptor) and *HPGD* (hydroxyprostaglandin dehydrogenase), have been reported to facilitate the conversion of human CD4^+^ T cells into induced Treg, or to maintain Treg suppressive functions, suggesting a potential acquired plasticity of these populations (49–51). Notably, the elevated gene profile was concentrated in the RNA-IL-22^+^ CD1a-restricted CD4^+^ T cells, but not in ADT-IL22^+^ population, implicating temporal regulatory mechanisms of gene expression in IL-22-producing T cells. IFNγ-producing CD4^+^ T cells displayed characteristics often associated with cytotoxic T lymphocytes (CTL), including high levels of inflammatory cytokines (*IFNG, CSF1* (M-CSF), *CSF2* (GM-CSF)), cytotoxic (*GZMA, GZMH, PRF1, NKG7, FASLG*), chemotactic (*CCL3, CCL4, CCL5, CXCR3, CCR5*), and transcription factor (*TBX21, Runx3*) signatures, as well as being enriched for enzymes and inhibitors promoting cytolytic activity (*CTSC* (cathepsin C), *CST7* (cystatin F), *APOBEC3G* (cytidine deaminase)) (Figure 3I). Several T cell exhaustion and inhibitory markers (*PDCD1, LAG3, HAVCR2, IL10)* were also elevated in these CD4^+^ CTLs (Figure 3I). Moreover, the majority of IFNγ-producing CD8^+^ T cells exhibited elevated T cell cytotoxicity, migration, activation, survival, and exhaustion-related genes (Figure 3J). Notably, this population displayed higher expression of MHC-II related genes (*HLA-DQA1, HLA-DQB1, HLA-DRB1, HLA-DRA, HLA-DPA1, HLA-DRB5, HLA-DPB1, CD74*) and genes related to cell cycling and division (*CDK6, CCND2, TYMS, ZBTB32, ADGRG1*), suggestive of highly proliferating T cell phenotypes (Figure 3J) (52).

We next identified differentially expressed immunophenotype markers for each CD1a-responding subgroup at the protein level. Each subgroup exhibited distinct molecular patterns which largely matched the RNA expression. CD1a-restricted CD4^+^ T cells expressed an array of activation markers (CD25 (*IL2RA*), CD71, OX40 (*TNFRSF4*), CD49d (*ITGA4*), 4-1BB (*TNFRSF9*)), chemokine receptors related to CTL (CXCR3) and Th17, and inhibitory/exhaustion markers (PD-L1, PD-1 (*PDCD1*), LAG-3, Tim3 (*HAVCR2*)) (Figure 3K). Similar activating and inhibitory patterns were observed in CD1a-restricted CD8^+^ T cells, with the additional expression of Natural Killer Cell Receptor 2B4 and CD94, and chemokine receptor CCR5 (Figure 3L). Consistent with the RNA results, HLA-DR was slightly elevated on IFNγ-producing CD1a-reactive CD8^+^ T cells (Figure 3L). Notably, some IFNγ-producing CD1a-reactive skin T cells displayed IL-2RB^+^ITGAE^+^CD69^+^ resident memory T cell (Trm) phenotypes (Figure S1I), indicating their roles in providing rapid tissue immune effector function. These results, together with the transcriptomic profiles observed, have characterized a diverse functionality of the IL-22- and IFNγ-secreting CD1a-reactive CD4^+^ and CD8^+^ T cells.

**Figure 4:**
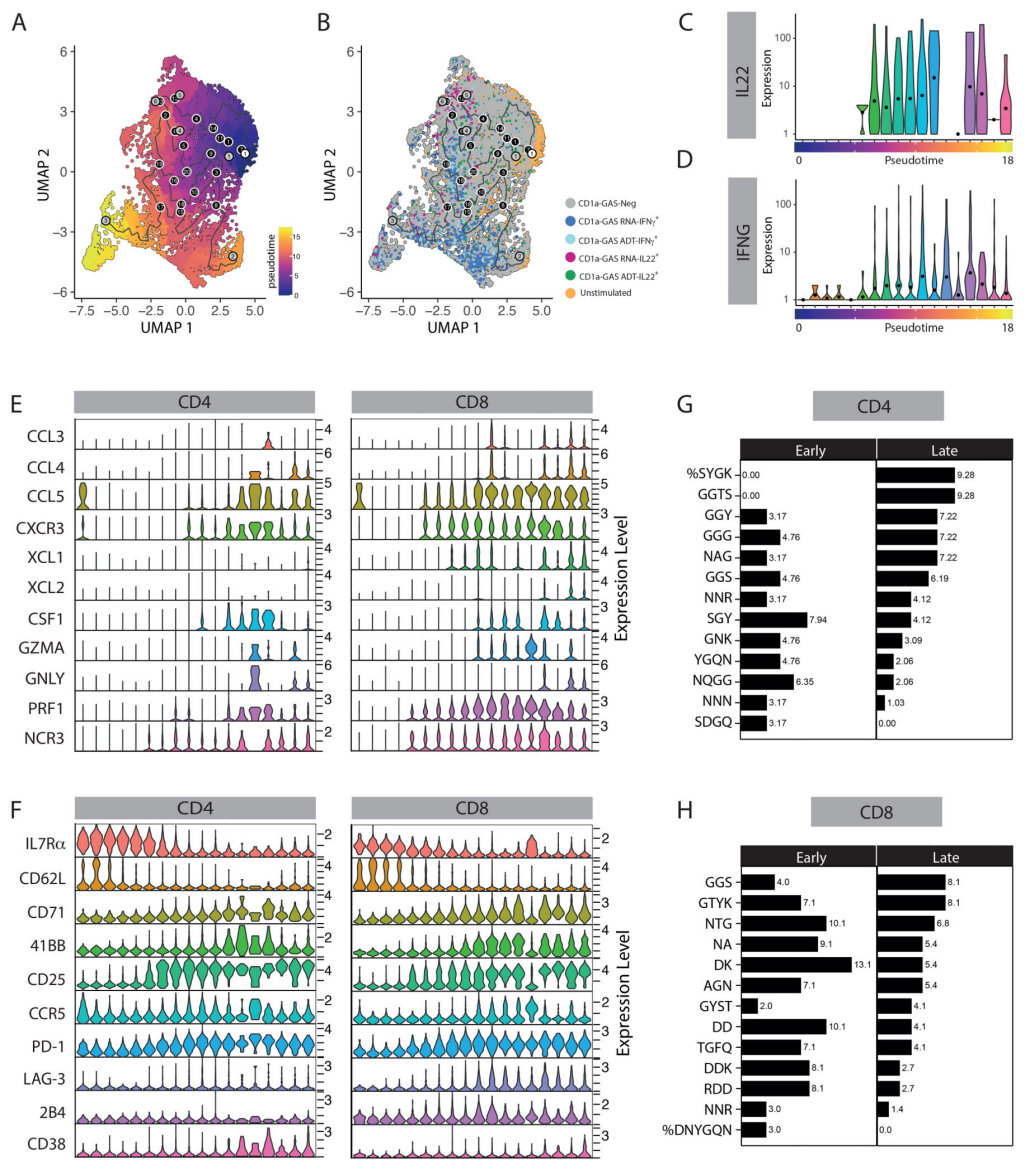
Pseudotime trajectory analysis depicts an effector gradient of skin T cells in response to CD1a-presentation. **(A)** Trajectory visualization of skin CD3^+^ cells after 6-hour co-culture with GAS-infected K562-CD1a cells (MOI=50). Cells were ordered and colored according to their pseudotime on UMAP plot. **(B)** UMAP plot capturing pseudotime progression of skin CD3^+^ cells by cytokine production. Violin plots showing IL-22 **(C)** and IFNγ **(D)** mRNA expression level changed over pseudotime trajectory. Violin plots demonstrating selective differentially expressed gene **(E)** and surface protein **(F)** expression patterns of the indicated markers in skin CD4^+^ and CD8^+^ T cells changed over pseudotime trajectory in response to CD1a-GAS presentation (genes with fold change ≥ 0.5, adjusted p < 0.05). Representative motif enrichment of CDR3α from CD4^+^ **(G)** and CD8^+^ **(H)** cells located at early and late pseudotime. The percentage of clonotypes containing each motif is indicated.

To understand the differentiation signatures of CD1a-reactive T cells, we constructed single-cell trajectories using the Monocle 3 R package (53); small clusters (with cell number < 165) 13, 14, 15 were removed and the remaining T cells were placed on the pseudotime trajectories based on changes in the transcriptomes (Figure 4A and Figure S2A), with unstimulated T cells assigned as the root node for ordering. The majority of unstimulated T cells and CD1a-GAS Neg (IL-22-IFNγ-) population distributed throughout the early pseudotime, whereas most of the IL-22- and IFNγ-producing subgroups were found in later pseudotime, showing a clear temporal separation (Figure 4B-D). We inspected the transition of expression values along the pseudotime for previously established genes associated with CD1a-restricted T cell activation, and found their expression to correlate with temporal development of T cell activation and differentiation. We identified a gradual increase in the expression of genes encoding chemotactic and cytotoxic molecules, as well as concordant expression of migration, inhibition, proliferation molecules, matching the progressive differentiation states of T cell effector functions (Figure 4E, S2B). Interestingly, after separation of CD4^+^ and CD8^+^ subsets, both CD4^+^ CTLs and CD8^+^ CTLs were found to be distributed in the latter half of the pseudotime (Figure 4E), showing a convergent differentiation pathway despite fundamental developmental differences between CD4^+^ and CD8^+^ T cells. Several genes were also found to be downregulated rapidly during the course of T cell activation, including genes essential for the homeostatic survival of naive T cells, such as PIK3IP1, GIMAP7, and IL7R (Figure S2C). Similar patterns of T cell activation were also observed on surface protein marker expression (Figure 4F). The data confirm that during activation, CD1a-reactive T cells follow a similar pattern of gene expression that has been observed for peptide-specific T cells, emphasizing an adaptive-like pathway in response to stimulation. Finally, we examined CDR3 residue composition using GLIPH2 software (54) to detect potential conserved CDR3 motifs with CD1a specificity. Expanded TCR clonotypes from cells located at early and late pseudotime were searched for enriched CDR3 motifs; and multiple CDR3α, but not CDR3β, motif candidates were identified within these T cell populations. The percentage of T cells containing those motifs, for both CD4^+^ and CD8^+^ populations, are shown in Figure 4G and 4H, respectively, showing differed motif preference in CD1a-reactive T cells located at late pseudotime. In summary, we describe a comprehensive phenotype of CD1a-reactive T cells during stimulation and show co-ordinated expression of activation and differentiation markers.

### Patients with psoriasis have elevated frequencies and activation of GAS-reactive T cells

**Figure 5:**
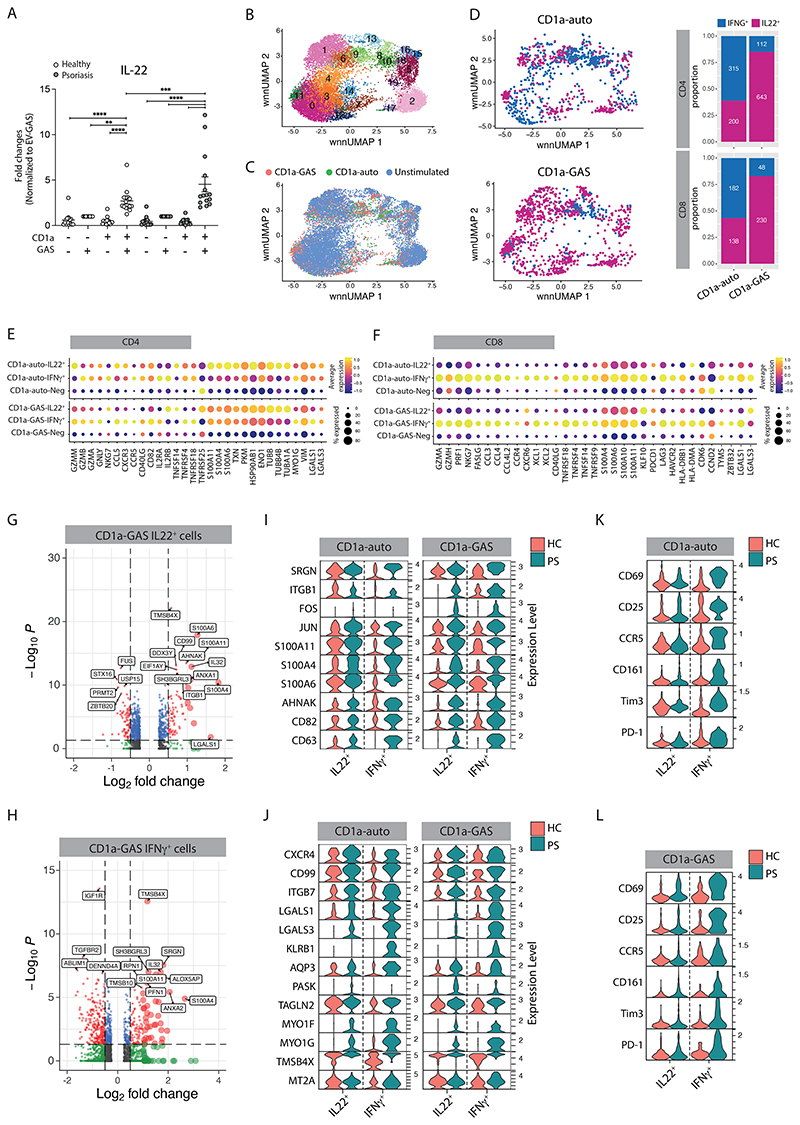
Psoriatic blood T cells show hyperreactivity in response to CD1a-related presentation. **(A)** Production of IL-22 from healthy (n=15) or psoriatic (n=15) polyclonal blood T cells detected by Secretion assay after 4-hour co-culture with control or GAS-infected K562 cells (MOI=50). Each symbol represents an individual (mean ± SEM). *P < 0.05, **P < 0.01 and ****P < 0.0001; two-way ANOVA with Tukey's post hoc test. Data are representative of more than three independent experiments. Single-cell multi-omic analysis of blood CD3^+^ cells isolated from five healthy and three individuals with psoriasis after 6-hour co-culture with unpulsed K562-CD1a (CD1a-auto) or GAS-infected K562-CD1a cells (CD1a-GAS). **(B)** UMAP plots showing unbiased clustering of the blood CD3^+^ cells. **(C)** UMAP plots showing the clustering of blood CD3^+^ cells according to the treatments (CD1a-auto, CD1a-GAS and unstimulated) **(D)** UMAP plots showing the clustering of IFNγ and IL-22-secreting cells (left panel) and their relative proportion within each co-culture condition (right panel). Dot plots showing the gene expression signatures of IL-22- and IFNγ-producing blood CD4^+^ **(E)** and CD8^+^ **(F)** T cells of healthy donors from CD1a-auto and CD1a-GAS treatments (selective genes with fold change ≥ 0.5, adjusted p < 0.05). Volcano plots showing differentially expressed genes in IL-22- **(G)** and IFNγ- **(H)** producing psoriatic CD4^+^ T cells, comparing to their healthy counterparts. The red symbols in volcano plots represent significantly upregulated or downregulated genes (fold change ≥ 0.5, adjusted p < 0.05). Only genes with ±0.25 log2 fold changes were shown on the Volcano plots. Violin plots demonstrate selective differentially expressed genes **(I-J)** and surface proteins **(K-L)** between psoriatic and healthy IL-22- and IFNγ-producing blood CD4^+^ T cells with indicated co-culture conditions (Genes or proteins with fold change ≥ 0.5, adjusted p < 0.05).

We and others have previously shown that patients with psoriasis exhibit aberrant release of type 22 and 17 related cytokines upon pan-T stimuli (Figure S3) (55), as well as elevated circulating and cutaneous CD1a-reactive T cells (29, 32), but there have been no studies which have addressed the relevance of GAS in the CD1a pathway in patients. Given we have demonstrated here that GAS can drive a CD1a-autoreactive effector T cell response, we next tested whether individuals with plaque psoriasis have altered frequency and phenotype of GAS-responsive T cells. Patients with psoriasis had significantly elevated frequencies of IL-22 producing circulating GAS-responsive CD1a-reactive T cells (Figure 5A). CD1a-autoreactive and GAS-responsive CD1a-reactive blood T cells producing IL-22, or IFNγ from five healthy and three psoriatic individuals were sorted and subjected to multiomic analysis. In total, 15,176 sequenced T cells passed quality control, doublet exclusion, and removal of *FOXP3*-expressing T cells. 19 clusters of T cell subsets and states were identified after UMAP visualization (Figure 5B), with cluster enrichment of T cells derived from each treatment condition as observed in the skin dataset (Figure 5C). In blood T cells, *IL-22* RNA expression level was only detected in 11 cells; hence we relied on both the RNA/protein expression and the sorting strategy of IL-22-producing populations to group the CD1a-responding cells into three subgroups: IL-22^+^, IFNγ^+^ and Neg (IL-22^-^IFNγ^-^). IFNγ-producing T cells generally formed distinct clusters from IL-22-producing cells, and as expected, while CD1a-autoreactive cells comprised both IL-22 and IFNγ producing cells, CD1a-GAS-reactive cells were predominantly IL-22-secreting (Figure 5D). Both naïve and antigen-experienced T cell subsets were found in the circulation (Figure S4A).

Blood IFNγ- or IL-22-producing CD1a-reactive T cells shared similar gene expression patterns to skin CD1a-reactive T cells. In short, CD1a autoreactive and GAS-reactive CD4^+^ and CD8^+^ T cells were highly active and proliferative, expressing genes related to T cell effector functions, cytoskeleton remodelling, cell adhesion, and metabolic programming (Figure 5E-F, Figure S4B). T cells with a naive phenotype equipped with the capacity of producing multiple cytokines (T_CNP_ cells) have been described in humans and mouse models (56, 57). Here, we observed that naïve blood T cells, concentrated in cluster 10 and 16, showed the ability to respond to CD1a presentation (Figure 5D, and S4A). Of note, as opposed to skin, some CD4^+^ IL-22-producing CD1a-reactive blood T cells, instead of displaying a Th17 phenotype, exhibited abilities to produce Th1 and cytolytic functionality (Figure 5E-F), suggesting a potential plasticity among blood Th subsets in response to the inflammatory milieu.

Next, we compared the phenotypes of IFNγ-producing or IL-22-producing CD1a-reactive T cells from healthy and psoriasis. The main DEGs were found within CD4^+^ T cell population (Figure 5G-H). To highlight, psoriatic CD1a-reactive T cells displayed higher characteristics of cytotoxicity with elevated expression of genes playing a role in formation of secretory granules (*SGRN*), and genes linked to their killing potential (*ITGB1*(CD29)) (Figure 5I) (58, 59), as well as transcription factors involved in TCR signalling (*FOS, JUN*) (60), and components in the TCR signalling cascade, including calcium-binding/signalling proteins, *S100A11, S100A4, S100A6*, and *AHNAK*, and co-stimulatory molecules *CD82* and *CD63* (Figure 5I). Furthermore, several of the transcripts encoded proteins with established roles in T cell chemotaxis, adhesion, tissue trafficking (*CXCR4, CD99, ITGB7, LGALS1, LGALS3, KLRB1*, and *AQP3*), as well as cell division and proliferation (*PASK, TAGLN2, MYO1F, MYO1G, TMSB4X, MT2A*) were also higher in psoriatic CD1a-reactive T cells (Figure 5J). *KLF6*, which has been reported to strongly associate with T cell activation in psoriasis, was also found to be expressed at higher levels in CD1a-GAS reactive IL-22-producing T cells (Figure S4C) (61). The hyperactive phenotype of psoriatic CD1a-reactive T cells was confirmed in surface protein profiles showing upregulated activation markers CD25 and CD69, tissue-associated markers CCR5 and CD161 (*KLRB1*) expression, and inhibitory markers Tim3 and PD-1(Figure 5K-L). Overall, these data show that individuals with psoriasis have higher frequencies of activated GAS-responsive CD1a-reactive T cells.

### GAS drives the activation of CD1a-reactive T cells

**Figure 6:**
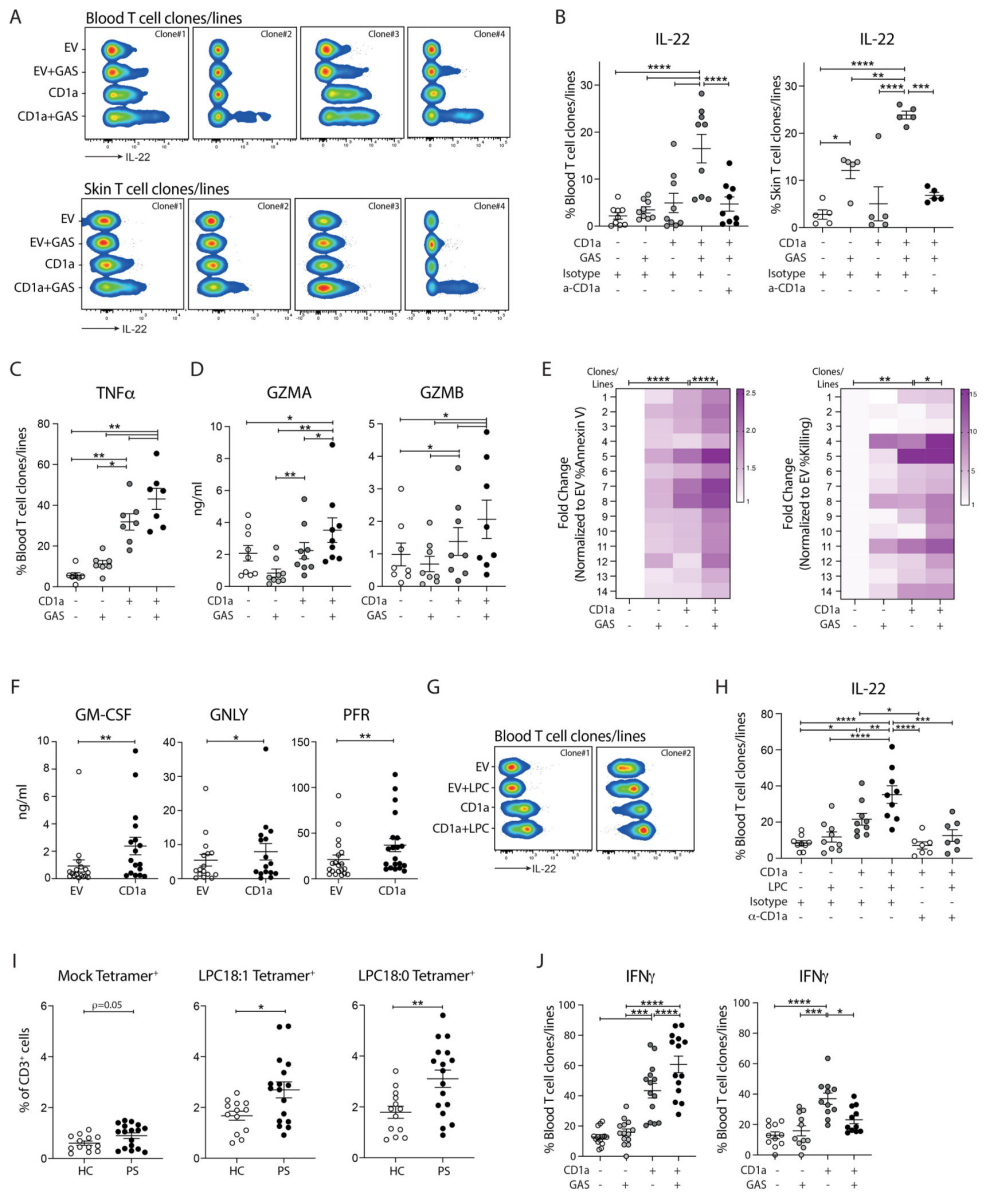
GAS drives the clonal expansion and activation of CD1a-reactive T cells with ability to lyse CD1a-expressing infected target cells. **(A-B)** Production of IL-22 from expanded blood or skin CD1a-reactive T cell clones/lines detected by Secretion assay after 4-hour co-culture with control and GAS-infected K562 cells (MOI=50). Anti-CD1a or isotype-matched control antibody (10 µg/ml) were added to block CD1a-specific activation. Four representative results are shown (n=5-9). **(C)** Production of TNFα from expanded blood CD1a-reactive T cell clones/lines detected by Secretion assay after 4-hour co-culture with control and GAS-infected K562 cells (MOI=50) (n=7). **(D)** Secretion of granzyme A (GZMA) and granzyme B (GZMB) from expanded blood CD1a-reactive T cell clones/lines analyzed by bead-based immunoassays after 24-hour co-culture with control and GAS-infected K562 cells (MOI=50) (n=8-9). **(E)** Flow cytometry analysis of the killing capacity of the blood CD8^+^ CD1a-reactive T cell clones/lines. The percentage of apoptotic cells (Annexin V^+^, left panel) and percentage of killing (right panel) result graph were calculated as the fold change of each condition to the K562-EV (n=14). **(F)** Secretion of GM-CSF, granulysin (GNLY) and perforin (PFR) from expanded blood CD1a-reactive T cell clones/lines analyzed by bead-based immunoassays after 24-hour co-culture with control and GAS-infected K562 cells (MOI=50) (n=16-20). **(G-H)** Production of IL-22 from expanded blood CD1a-reactive T cell clones/lines detected by Secretion assay after 4-hour co-culture with control or LPC-pulsed K562 cells (150 µM). Anti-CD1a or isotype-matched control antibody (10 µg/ml) were added to block CD1a-specific activation. Two representative results are shown (n=9). **(I)** CD1a tetramer staining of CD3^+^ T cells in a cohort of 13 healthy controls and 17 PS patients. Percentages of indicated tetramers^+^ cells among all T cells analyzed by flow cytometry. Each symbol represents an individual donor (mean ± SEM). **(J)** Production of IFNγ from expanded blood CD1a-reactive T cell clones/lines detected by Secretion assay after 4-hour co-culture with control and GAS-infected K562 cells (MOI=50) (n=14, left panel; n=11, right panel). Each symbol represents a T cell clone/line (B, C, D, F, H, J) (mean ± SEM). *P < 0.05, **P < 0.01, ***P < 0.001 and ****P < 0.0001; two-way ANOVA with Tukey's post hoc test (B, H), repeated-measures (RM) one-way ANOVA with Tukey's post hoc test (C, D, E, J) or two-tailed paired t test (F, I). Data are representative of more than three independent experiments.

In order to investigate the underlying mechanisms, we sorted IL-22-producing GAS-reactive T cells using flow cytometry and went on to successfully establish T cell clones/lines derived from blood and skin. The blood and skin clones/lines were able to recognize GAS-infected K562-CD1a cells (Figure 6A), and this could be inhibited by anti-CD1a blockade (Figure 6B). The isolated clones/lines were unable to show enhanced recognition of K562-CD1a infected with other streptococcal and staphylococcal species with importance to the skin and other epithelial barrier surfaces, suggesting that the CD1a pathway is particularly relevant to *Streptococcus pyogenes* of those tested (Figure S5A-B). Clones/lines could be either CD4^+^ or CD8^+^, but all maintained their ability to produce IL-22 in response to GAS-infected K562-CD1a cells. Interestingly, some T cell clones/lines could also recognize uninfected K562-CD1a cells, suggesting their potential CD1a-autoreactivity and this was explored next.

### CD8^+^ GAS-reactive T cells lyse infected and uninfected target cells

Given the single cell *ex vivo* data showing evidence of cytolytic potential within the GAS-reactive CD8^+^ T cells, we next tested whether the CD8^+^ subset had cytolytic function. Streptococcus is known to bind to Langerhans cells, and can infect mononuclear phagocytic cells and epithelial cells (62–64). We first confirmed the mRNA signal observed in the *ex vivo* single cell analysis and showed production of TNFα, granzyme A (GZMA), and granzyme B (GZMB) at the protein level in response to GAS-infected target cells (Figure 6C-D). Furthermore, the clones/lines were able to lyse GAS-infected target cells implicating a role of the CD8^+^ T cells in death of infected cells (Figure 6E, S6). Interestingly, the CD8^+^ T cell clones/lines could also recognize uninfected K562-CD1a target cells (Figure 6C-E), and produce high quantities of GM-CSF, granulysin (GNLY) and perforin (PFR) (Figure 6F). These data show that many GAS-reactive CD8^+^ T cells can lyse CD1a-expressing GAS-infected target cells, but can also show autoreactivity, with implications for effector function at uninfected sites and mechanisms underlying immunopathology of post-streptococcal disease. Given the autoreactivity of the GAS-driven T cells, we next explored candidate self-lipid antigens for recognition by this sub-population of T cells.

### A proportion of GAS-reactive T cells can respond to the self-lipid lysophosphatidylcholine

We have previously shown that endogenous and exogenous phospholipases (PLA_2_) can generate lipid antigens for recognition by CD1a-reactive T cells (29, 37, 38). Furthermore, the PLA_2_ lipid products lysophosphatidylcholine (LPC) and oleic acid are known permissive CD1a ligands (34, 38). It is of interest that *Streptococcus pyogenes* expresses PLA_2_ activity which participates in host pathogen interaction and is a virulence factor (65, 66). Here we show that a proportion of the GAS-driven T cell clones/lines recognize LPC-pulsed K562-CD1a cells (Figure 6G-H). These data suggest that GAS infection is in part detected by recognition of self-lipid antigens, which are products of the PLA_2_ pathway.

Recent advances in CD1a tetramer technology facilitate the identification of CD1a-reactive T cells recognizing specific lipids (36, 67). To detect the frequency of CD1a-LPC-reactive T cells in individuals with plaque psoriasis, we next tetramerized CD1a monomers treated with CHAPS detergent (mock) or different species of LPCs (Figure S7A), and stained polyclonal blood T cells. A significantly higher frequency *ex vivo* of circulating CD1a-LPC-tetramer-binding T cells (Figure 6I and S7B-C)) were identified in these patients, in line with our finding of elevated GAS-reactive CD1a-restricted effector T cell responses in psoriatics (Figure 5). Overall, the data suggest that GAS can drive the activation of CD1a-autoreactive T cells which can respond to skin stress lipids, including LPC, implicating their involvement in psoriatic immunopathology.

It was noted that not all the GAS-driven T cells recognized K562-CD1a cells in the absence of GAS, and so it is likely that there are other bacterial-specific ligands recognized by other GAS-reactive T cells. Therefore, while the IFNγ-producing CD1a-reactive T cell frequency was not altered in net *ex vivo* polyclonal T cells in response to GAS-infection (Figure 2E), we tested whether this might mask individual patterns of GAS-reactivity at the clonal T cell level. We sorted and clonal expanded IFNγ-producing CD1a-autoreactive T cell lines from blood. Surprisingly, approximately half of the IFNγ-producing CD1a-autoreactive T cell lines recognized GAS-pulsed K562-CD1a cells (Figure 6J left panel), whereas the autoreactivity of other T cell lines was inhibited by GAS-derived ligands (Figure 6J right panel), which supports the possibility of other bacterial-specific CD1a ligands generated during GAS infection. Overall, these data identify permissive self-lipid LPCs as a potential target for subsets of GAS-reactive T cells.

### CD1a-reactivity is TCR-dependent

**Figure 7:**
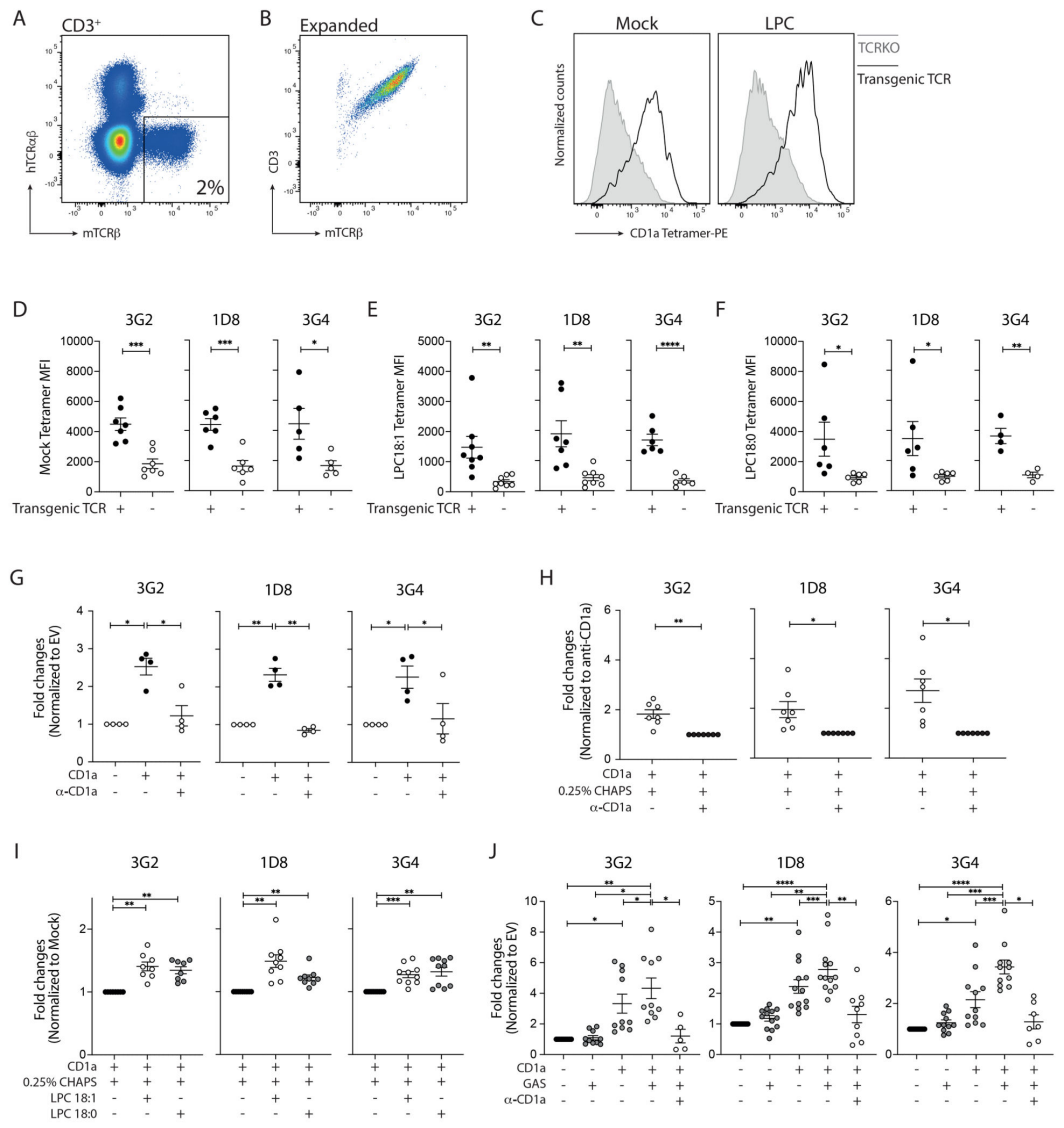
TCRs derived from GAS-responsive CD1a-autoreactive T cell clones render CD1a-lipid specificity. **(A)** Representative image showing the successful replacement of the endogenous TCR with transgenic TCR expressing mouse constant region. **(B)** Representative image showing the purity of the expanded transgenic TCR expressing T cells. PBMCs from multiple donors were engineered and sorted per target TCR. **(C)** Representative images of TCR-transgenic T cells stained with mock-treated or LPC-treated CD1a tetramers. **(D-F)** Mean fluorescence intensity (MFI) of indicated CD1a tetramer on each TCR-transgenic T cells (n=4-8). **(G)** Production of intracellular Cytokine (IFNγ- or GM-CSF)-from expanded TCR-transgenic T cells analyzed by flow cytometry after 4-hour co-culture with K562 cells. Anti-CD1a or isotype-matched control antibody (10 µg/ml) were added to block CD1a-specific activation. The overall data were graphed as the fold change of each condition to the CD1a blockade condition (n=4). **(H)** Cytokines (IFNγ- or GM-CSF) release from TCR-transgenic T cells co-cultured with bead-bound CD1a treated with 0.25% CHAPS (mock) measured by intracellular staining and analyzed by flow cytometry after 4-hour co-culture. The overall data were graphed as the fold change to the CD1a blockade condition (n=7). **(I)** Cytokines (IFNγ- or GM-CSF) release from TCR-transgenic T cells co-cultured with bead-bound CD1a treated with indicated lipids measured by intracellular staining and analyzed by flow cytometry after 4-hour co-culture. The overall data were graphed as the fold change to the mock condition (n=8-10). **(J)** Production of intracellular Cytokine (IFNγ- or GM-CSF)-from expanded TCR-transgenic T cells analyzed by flow cytometry after 4-hour co-culture with control or GAS-infected K562 cells (n=10-13). Anti-CD1a or isotype-matched control antibody (10 µg/ml) were added to block CD1a-specific activation. Each symbol represents a T cell clone/line (mean ± SEM). *P < 0.05, **P < 0.01, ***P < 0.001 and ****P < 0.0001; two-tailed unpaired t test (D, E, F), two-tailed paired t test (H), repeated-measures (RM) one-way ANOVA with Tukey's post hoc test (G, I), or mixed-effects one-way ANOVA with Tukey's post hoc test (J). Data are representative of more than three independent experiments.

To address the TCR-mediated CD1a reactivity, we sequenced the TCRs of three GAS-responsive CD1a-autoreactive T cell clones (3G2, 1D8 and 3G4), and undertook TCR gene transfer experiments using CRISPR-Cas9 editing to orthotopically replace endogenous TCRs with target TCRs via homology-directed repair (HDR). T cells engineered with the transgenic TCRs were isolated and expanded (Figure 7A-B), and were able to bind to CD1a-mock (detergent-treated CD1a) and CD1a-LPC tetramers (Figure 7C-F), as well as recognize CD1a-expressing K562 cells (Figure 7G) or bead-bound CD1a proteins treated with CHAPS detergent (mock) (Figure 7H). Moreover, when TCR-transgenic T cells were co-cultured with bead-bound CD1a proteins treated with synthetic LPC species, further increases in cytokine production were detected (Figure 7I), suggesting that the T cells could respond to CD1a which was enhanced in the presence of the permissive ligand LPC. Furthermore, the engineered T cells were able to recognize GAS-infected K562-CD1a cells in a CD1a-dependent manner (Figure 7J). Altogether, the data show that the CD1a-reactivity of the T cell clones is TCR-dependent and confirms reactivity to mock CD1a and enhanced with the CD1a loaded with the self-lipid LPC.

### GAS infection drives a CD1a-dependent psoriasis-like inflammatory response in vivo

**Figure 8:**
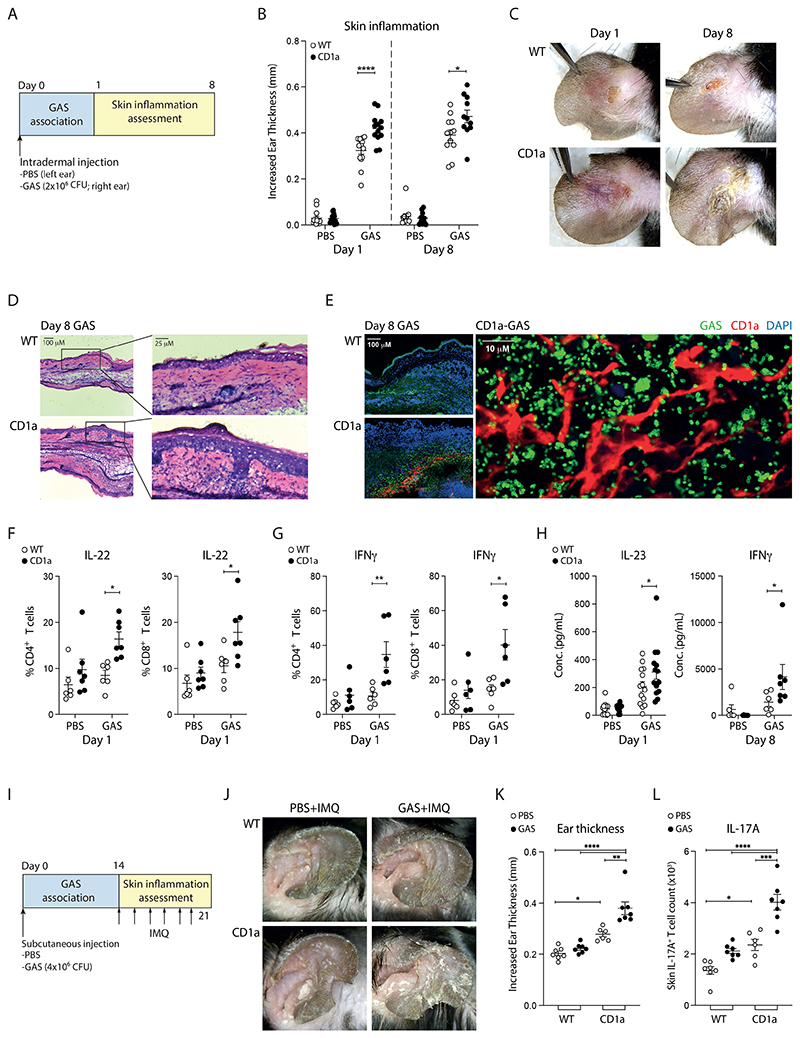
GAS exacerbates skin inflammation through CD1a *in vivo*. **(A)** Schematic of GAS-induced skin inflammation. **(B)** Measurement of ear swelling induced by GAS-infection of wild-type (WT) and CD1a transgenic mice (CD1a) at day 1 and day 8 (n=12-14). **(C)** Representative images of inflammation on day 1 and 8 of the GAS-infection of WT and CD1a transgenic mice. **(D)** Microscopy of hematoxylin and eosin-stained cross sections of ears from mice infected with GAS for 8 days. **(E)** CD1a and GAS within ear skin of WT and CD1a transgenic mice 8 days after GAS infection were visualised by immunofluorescence (DAPI (blue), anti-CD1a (red) and anti-GAS (green)). **(F-G)** Intracellular staining analysis of T cell cytokines in draining lymph nodes from mice infected 1 day after GAS infection (n=6-7). **(H)** Concentrations of IL-23 and IFNγ in ear skin extracts of GAS-infected WT and CD1a transgenic mice were analyzed by bead-based immunoassays after 1-day and 8-day GAS inoculation (n=6-14). **(I)** Schematic of IMQ-induced skin inflammation post GAS infection. **(J)** Representative images of psoriasiform inflammation on day 7 of the IMQ treated WT and CD1a transgenic mice with or without prior GAS infection. **(K)** Day 7 measurement of ear swelling induced by IMQ treatment of wild-type (WT) and CD1a transgenic mice (CD1a) with or without prior exposure of GAS (n=6-7). **(L)** IL-17A-producing T cell counts per ear by intracellular staining of the IMQ treated WT and CD1a transgenic mice with or without prior GAS infection (n=6-7). Each symbol represents an individual mouse (mean ± SEM). *P < 0.05, **P < 0.01 and ****P < 0.0001; two-way ANOVA with Tukey's post hoc test (B, F, G, K, L), or two-way ANOVA with Šídák's post hoc test (H). Data are representative of more than three independent experiments.

CD1a presents both endogenous and exogenous lipid antigens to activate T cells in human CD1a transgenic (CD1a-Tg) challenge models, including sensitization of mice skin with urushiol, a sap compound found in poison ivy, and imiquimod, a TLR7 agonist that can trigger psoriasiform inflammation (32, 68). To investigate whether GAS can exacerbate skin inflammation through CD1a *in vivo*, we intradermally challenged CD1a-Tg and wild-type mice with live GAS to the ear skin and the skin inflammation was assessed at day 1 and day 8 post-infection (Figure 8A). Ear thickness was significantly increased after GAS infection and advanced further in the presence of CD1a (Figure 8B). The appearance of a typical lesion progression after GAS infection showed marked erythema and scaling and expanded lesion site in the CD1a-Tg mice (Figure 8C). Histological analysis revealed an increased thickening of both epidermis and dermis of GAS-infected CD1a-Tg mice compared to wild-type mice, with increased rete ridge prominence, which are known features of psoriatic inflammation (Figure 8D). Confocal fluorescence microscopy analysis of skin showed the infiltration of CD1a-expressing cells and their proximity to GAS, suggesting the near-neighbour potential of antigen processing and presentation (Figure 8E).

We analyzed the skin, draining lymph node and spleen cells by flow cytometry and found a significant increase in lymphocytes, neutrophils and monocytes after GAS-infection but the relative proportions of the immune cells were not significantly different observed between wild-type and CD1a-Tg mice (Figure S8). However, the presence of CD1a promoted the production of IL-22 and IFNγ from both CD4^+^ and CD8^+^ draining lymph node T cells after GAS infection (Figure 8F-G). Cytokine profile analysis of skin extracts also showed an overall inflammatory myeloid related cytokine upregulation after GAS infection (Figure S9); we observed an increased concentration of IL-23, which plays an essential role in type 17 pathway induction in psoriasis, at day 1, and elevated IFNγ level at day 8 (Figure 8H).

We next investigated the longitudinal effects of GAS infection in CD1a-Tg mice using a distal application of imiquimod (IMQ) model (68). We subcutaneously challenged CD1a-Tg and wild-type mice with live GAS to the back, and after GAS clearance (Day 14) daily topical skin application of IMQ cream on the distal ear skin induced significant psoriasiform inflammation (Figure 8I-J). CD1a-Tg mice with prior GAS infection displayed more pronounced pathological changes of scaliness, erythema, and significantly increased ear thickness (Figure 8J-K). An increase in IL-17A-producing T cells in the ear skin of the CD1a-Tg mice was identified in GAS-experienced CD1a-Tg mice (Figure 8L). In addition, T cells-derived from skin-draining lymph nodes and spleen of IMQ/GAS-treated CD1a-Tg mice exhibited enhanced responsiveness to CD1a presentation *in vitro* (Figure S10). Collectively, the mouse *in vivo* models have provided evidence of a role of GAS in driving psoriatic skin inflammation which is enhanced in the presence of CD1a.

## Discussion

Post-streptococcal inflammatory disease has long been known in the clinic, but mechanisms addressing the underlying pathogenesis have not been fully elucidated. We have shown that GAS-responsive CD1a-reactive T cells comprise a substantial portion of the human αβ T cell repertoire, accounting for up to 5-10% of T cells. Subsets of the CD1a-reactive cells which activate and proliferate in response to GAS, are able to also respond to the self-lipid antigen LPC, a known PLA_2_ product present in the skin. This suggests that GAS can drive a CD1a-dependent auto-reactive T cell response, allowing LPC to act as a signal of tissue damage in response to bacterial infection.

In contrast to common pharyngeal intracellular presence, Group A streptococci are detected at low levels in healthy and psoriatic skin and blood (69, 70). Given published literature and the data shown here, during active GAS pharyngeal infection, it is likely that CD1a-autoreactive T cells will proliferate, activate, and acquire skin homing receptor expression. Therefore, through mounting a local tissue response, it is conceivable that GAS will have the potential to drive ensuing CD1a-dependent cutaneous inflammation.

The CD1a-reactive T cells were found to produce IL-22, which is known to be elevated in psoriatic skin lesions and serum levels correlate with disease activity (71). Furthermore, IL-22 can promote keratinocyte proliferation and production of antimicrobial responses (71, 72). It is of interest that IL-22 is also elevated in wounds, and psoriasis lesions can show the Koebner phenomenon where disease develops at sites of skin trauma. LPC is produced during platelet activation at wounds, and elevated levels of LPC are detectable in lesional psoriatic skin (73). In addition, CD1a is acquired by large numbers of infiltrating dendritic cell populations infiltrating skin wounds (25, 74, 75). It is therefore possible that a similar mechanism could contribute to forms of sterile inflammation such as the Koebner phenomenon.

It was noted that many of the CD1a-autoreactive T cells were CD8^+^ and had cytolytic activity. Such cells may have the capacity to kill infected cells *in vivo*, reducing the intracellular GAS reservoir within CD1a-expressing cells. Intracellular residence would provide an advantage to GAS as there would be relative protection from neutrophil degranulation, antibody-, and complement-mediated inhibition and some antibiotic effects (76). A CD1a-dependent mechanism of killing would provide the immune system with an alternative strategy for bacterial reservoir control that would depend less on relatively inefficient cross-presentation pathways. However, it was also noted that the CD8^+^ T cells could lyse uninfected CD1a-expressing targets confirming that bacterial-driven T cell reactivity can drive a CD1a-dependent autoreactive response and associated inflammation.

On the basis of our current understanding of MHC-peptide recognition by T cells, it might be predicted that there would be diverse lipid antigens for recognition. While this is likely to be true, the data suggest that under inflammatory conditions, subsets of the GAS-responsive CD1a reactive T cells can recognize broad families of permissive self-lipids, of which LPC was studied here. These data and published data (33) challenge the exquisite antigen-specific discrimination accepted within MHC-peptide dogma, where under inflammatory conditions, the diverse GAS-responsive CD1a-reactive T cells can be co-opted to control GAS infection through recognition of skin self-lipids. Such a system must be tightly controlled which may include spatial separation, inhibitory receptor and inhibitory lipid expression (67) and the nature of the local inflammatory milieu. It is likely that other layers of local control will be deployed, including a role for regulatory T cells. Nevertheless, it is clear that many of the CD1a-reactive T cells that are induced by GAS infection can respond to the self-lipid LPC and drive autoreactivity.

By linking GAS infection to the CD1a pathway, the data presented point to a wider interpretation of post-streptococcal disease in which GAS drives autoimmunity across different tissues. While this may be mediated through differential pathways at different sites, the findings identify non-peptide self-ligands as of broader relevance, and extend the Gell and Coombs classification which implicates a requirement for haptenation of non-peptide ligands. Given that CD1a is relatively non-polymorphic, this raises the possibility that broadly applicable therapeutics targeting CD1a may be feasible. Psoriasis is very common, affecting up to 2% of the population, suggesting there may be selection advantages, perhaps related to cutaneous immunity. The findings presented here would be compatible with the possibility that the GAS-induced CD1a-autoreactive T cells contribute to the GAS-specific immune response but at a cost of increased risk of psoriatic disease. This has relevance for understanding of fundamental biology related to bacterial-associated inflammation, but also in terms of capitalizing on a therapeutic window before the inflammatory sequelae ensue.

## Materials and methods

### Study design

The objective of this study was to determine the involvement of CD1a pathway in the pathogenesis relevance of post-streptococcal sequalae. We assessed the frequencies and functionalities of the CD1a-restictive GAS response circulating and cutaneous T cells from healthy individuals or psoriasis patients, using single-cell CITE-seq, T cell clonal expansion, and orthotopic TCR replacement. Randomization was not required due to the lack of intervention and blinded assessment of results was not performed. Clinic participants were only excluded if on systemic immunosuppression. Inter donor variation of functional responses was expected, because of the age, gender, ethnicity, and medical history of the individual recruited. Transgenic mice were used for *in vivo* GAS infection experiments, with approved humane end points. Animals were age matched and randomly assigned, and the studies were unblinded. The number of samples/donors/animals and the number of independent experiments are indicated in the figure legends. Sample size was determined on the basis of previous studies (25, 29, 37, 68).

### Cell lines

Empty vector-transfected K562 (K562-EV) and CD1a-transfected K562 (K562-CD1a) cells (a gift from B. Moody, Brigham and Womens Hospital, Harvard Medical School, Boston, MA) were maintained in R10 (RPMI 1640 medium supplemented with 10% FCS, 100 IU/ml penicillin, 100 μg/ml streptomycin (Gibco), 2 mM L-glutamine (Gibco), 1X nonessential amino acids (NEAAs) (Gibco), 1 mM sodium pyruvate (Gibco), 10 mM HEPES (Gibco), 50 μM 2-mercaptoethanol (Gibco)), and 800 μg/ml G418 antibiotic (Thermo Fisher Scientific).

### Bacterial strains and culture conditions

Streptococcus pyogenes (GAS) serotype M18 strain (ATCC® BAA‐572TM) was collected in the United States in 1987. The following reagent was obtained through the NIH Biodefense and Emerging Infections Research Resources Repository, NIAID, NIH as part of the Human Microbiome Project: Staphylococcus epidermidis (Strain BCM0060; HM-140), Streptococcus mitis (Strain F0392; HM-262), and Streptococcus pneumoniae (Strain TCH8431; HM-145). All bacteria strains were preserved in 10% glycerol stock and stored at -80°C. The frozen bacteria strains were streaked onto Columbia horse blood agar plates (OXOID) and cultured overnight at 37°C in a humidified 5% CO_2_ incubator. Colonies were collected and resuspended in DPBS (no calcium, no magnesium) before their use in infection experiments. To obtain bacteria culture at log phase of growth, bacteria were grown in Todd-Hewitt broth (Sigma-Aldrich) overnight in 5% CO_2_ at 37°C without shaking. The culture was pelleted by spinning at 2,500 rpm and resuspended in DPBS (no calcium, no magnesium). Heat-killed bacteria were generated by incubating the bacteria at 65°C for 10 min.

### Isolation of human blood and skin T cells

Human blood samples were obtained from healthy or individuals with plaque psoriasis, and skin samples were obtained from healthy donors undergoing plastic surgery. The individuals with psoriasis did not have arthritis and were not on systemic therapy. All specimens were taken under good clinical practice guidance with ethical approval (14/SC/0106, National Research Ethics Service [NRES]). Clinical metadata of psoriasis patients are shown in Supplementary table I. Peripheral blood mononuclear cells (PBMCs) were isolated using Lymphoprep (Stem Cell Technologies) gradient isolation. Skin samples were dissected and incubated with 1 mg/ml collagenase P (Roche) overnight at 37°C with 5% CO_2_. The next day 100 µg/ml DNase I (Roche Diagnostic) was added for 15-30 min. Cold 10 mM EDTA solution was then added to the sample to stop the digestion. The digested tissue was passed through a 70-µm cell strainers, and mononuclear cells were harvested with Lymphoprep gradient isolation before further procedures. Blood and skin T cells were isolated using Magnetic-activated cell sorting with CD3 MicroBeads (Miltenyi Biotec) following the manufacturer’s protocol, and resting in TCM (RPMI medium supplied with 10% HS, 100 IU/ml penicillin, 100 μg/ml streptomycin (Gibco), 2 mM L-glutamine (Gibco), 1X nonessential amino acids (NEAAs) (Gibco), 1 mM sodium pyruvate (Gibco), 10 mM HEPES (Gibco), 50 μM 2-mercaptoethanol (Gibco)) and IL-2 (200 IU/ml; BioLegend) for 72 h.

### Secretion assay

K562-EV or K562-CD1a were pulsed with GAS (MOI=50 or 100) for 72 hours and the extracellular bacteria were removed before coculturing with T cells. In some conditions, K562-EV or K562-CD1a cells were pulsed with lysophosphatidylcholine 18:1 (150 µM; Avanti Polar Lipids) for 16 hours and the excess lipids were removed before coculturing with T cells. Blood or skin T cells (1x10^6^) were co-cultured with control/pulsed K562-EV or K562-CD1a (0.5x10^6^) for 4-6 hrs. In indicated conditions, K562-CD1a was pretreated with anti-CD1a blocking antibody (10 µg/ml) or IgG1 isotype control (10 µg/ml; BioLegend) for 1 hr before the addition of T cells. Cytokine producing responder T cells were detected using Cytokine Secretion assays (Miltenyi Biotec) following the manufacturer’s instructions. T cells were coated with anti-cytokine (IL-22, IFNγ, GM-CSF, or IL-17A) antibody after coculture to detect CD1a dependent autocrine cytokine production using fluorochrome-conjugated detection antibodies. Antibodies against surface markers identifying T cells (anti-CD3, anti-CD4, anti-CD8, anti-TCRαβ) and their phenotypes (anti-CD45RA, anti-CD45RO, anti-CD25, anti-CD69, anti-CD137, anti-CD154, anti-CLA). Data were acquired using LSRFortessa X-50 flow cytometer (BD Biosciences) and further analyzed with FlowJo (FlowJo LLC) software.

### Sample preparation, CITE-seq staining and single cell RNA-seq

Skin T cells from four healthy individuals and blood T cells from five healthy and three psoriatic (plaque psoriasis) donors were subjected to single cell multiomic analysis. To construct a dataset comprising GAS-responsive CD1a-reactive T cells, we adapted our previous K562-CD1a stimulation strategy. PE- or APC-conjugated detection antibodies were used to detect IL-22- or IFNγ-producing T cells, respectively. After the FACS antibodies staining step of T cell Secretion assay, cells were incubated with FcX block (BioLegend) for 10 min, and stained with TotalSeq-C antibody pool (Supplementary table II) and a unique hashtag for each sample at 4°C for 30 min. Cells were then washed 3 times in staining buffer (0.4% BSA in PBS) and filtered using a 40 µm Flowmi filter (Sigma-Aldrich) and pooled in equal proportions. IL-22-producing, IFNγ-producing and non-IL-22/IFNγ-producing T cells after co-cultured with K562-CD1a or GAS-infected K562-CD1a were sorted. *Ex vivo* isolated unstimulated T cells were included to establish phenotypic baseline. Cells were loaded into 9 lanes of two 10x Genomics Chip G, at 20-30,000 cells per lane using a Chromium Single Cell Controller (10x Genomics, Pleasanton, CA) with the Chromium Single Cell 5’ Library & Gel Bead Kit v1.1. Remaining steps were carried out according to the manufacturer’s instructions and Cell Surface Protein/Immune Receptor Mapping Libraries and 5′ Gene Expression (GEX) Libraries were generated. Final libraries were sequenced on a NovoSeq 6000 (Illumina, San Diego) to achieve an average depth of 5, 000 raw reads per cell for CITE-seq Libraries and 25,000 raw reads per cell for GEX Libraries.

### Data processing, alignment, quality control, and hashtag demultiplexing of single cell RNA-seq

For each sequenced scRNA-Seq pool, Cell Ranger toolkit (version 6.0.1; 10X Genomics; https://support.10xgenomics.com/single-cell-gene-expression/software/downloads/latest) was used to process raw data, map cDNA libraries against hg38 human reference genome from the UCSC ftp site (77) and to summarize unique molecular identifier (UMI) counts against the corresponding Ensemble gene annotations (78). Hashed feature count matrix was CLR (Centered Log-Ratio) normalized and demultiplexed based on their sample of origin using R package Seurat’s HTOdemux function. In brief, normalized counts for each hash ID were fitted with a negative binomial distribution. Positive threshold was set to 99th percentile of the recovered normalized UMI counts for the hashtag where cells below this threshold was considered negative for the tag. Cells negative for hashtags and cells positive for multiple hashtags were filtered out. After filtering out and assigning the cells of origin based on HTO staining, we further removed the cells with less than 200 or greater than 4,000 detected genes, less than 1% or greater than 10% mitochondrial reads per each library. With 13 mitochondrial and 104 ribosomal genes which were highly variable among samples, genes that were expressed in <10 cells were removed from the final count matrix. Total number of UMI count per cell, percentage of mitochondrial features, individual donor effect was regressed out during the library merging. In total, 14,732 sequenced effector T cells passed quality control, doublet exclusion, and removal of FOXP3-expressing populations. Further detail can be found in supplementary methods.

### CD1a-reactive T cell clone/line generation and activation analysis

CD1a-restricted T cells were isolated by fluorescence activated cell sorting after co-culture with non-infected/infected K562-EV or K562-CD1a. The live responder cells were then single-cell sorted into a 96-well U-bottom culture plate and expanded with mixed lymphocyte reaction. The expanded T cell clones/lines were then check for purity and CD1a-responsiveness using Cytokine Secretion Assays (Miltenyi Biotec). Briefly, non-infected/infected K562-EV/CD1a (2x10^5^) were co-cultured with 1-5x10^5^ CD1a-reactive T cell lines/clones for 4 hrs with the addition of helper cytokines to support CD1a-dependent cytokine production: IL-12 (1 ng/mL; BioLegend), IL-18 (1 ng/mL; BioLegend) and IL-2 (25 U/mL; BioLegend) for IFNγ-producing T cells, and IL-6 (5 ng/mL; BioLegend), TNF-α (5 ng/mL; BioLegend), and IL-2 (25 U/mL; BioLegend) for IL-22-producing T cell culture. Supernatant was collected and stored at -80°C.

### FACS based Cytotoxicity assay

Target K562-EV/CD1a cells were fluorescently labelled with CellTraceViolet (Invitrogen) prior to the infection. CD1a-restricted T cell lines/clones (1-5x10^5^) were added to non-infected/infected target K562-EV/CD1a cells (2x10^5^) in the presence of IL-12 (1 ng/mL; BioLegend) and IL-18 (1 ng/mL; BioLegend) and IL-2 (25 U/mL; BioLegend). Supernatant was collected after 24 hr co-culture for cytokine analysis. Cell death was assessed by flow cytometry after 48 hrs co-culture. Briefly, the wells were harvested, and to stain for dead and apoptotic cells, Zombie Fixable Viability dyes (1:1000; BioLegend) and Annexin V-APC (BioLegend) were added. To allow quantitative analysis of the target cell populations, 2x10^5^ CFSE-labelled K562 cells (as reference cells) were added. This was done just prior to the FACS analysis to avoid the interaction between the target, reference, and T cells. Data were acquired using LSRFortessa X-50 flow cytometer (BD Biosciences) and further analyzed with FlowJo (FlowJo LLC) software. The percentage of induced killing was then calculated with the following equation by comparing the frequency of live target and reference populations: % cytotoxicity = 100-((% live target cells /% live reference cells)/(% live cells of untreated K562-EV/% live reference cells) x 100).

### CD1a tetramer staining

Biotinylated human CD1a monomers (NIH Tetramer Core Facility) were produced in HEK293-derived cell lines (36, 67). CD1a (10 ug) was treated with a 100X molar excess of LPC 18:1 or LPC 18:0 (Avanti Polar Lipids) in Tris Buffer saline containing 0.25% CHAPS or vehicle alone (mock) for 16 h at 37 °C, and tetramerised with PE Streptavidin (High Concentration; BioLegend) at a molar ratio of 5:1. T cells (<1x10^6^) were washed twice in FACS staining buffer (BioLegend) at room temperature and stained with 0.5 µl tetramer in 20 µl FACS staining buffer at 37 °C for 30 min with gentle shaking. Anti-CD3 antibody (OKT3; 0.1 µg in 10 µl; BioLegend) was added to the cells and incubated for an additional 10 min at 37 °C with gentle shaking. Tetramers and anti-CD3 antibody were removed before staining surface markers CD3 (UCHT1; BioLegend), CD4, CD8 and Zombie Fixable Viability dyes (BioLegend) for 15 min at 4°C. Cells were washed once and resuspended in FACS buffer and ready for requisition using LSRFortessa X-50 flow cytometer (BD Biosciences) and further analyzed with FlowJo (FlowJo LLC) software.

### Homology-directed repair (HDR) DNA template design

DNA templates were designed in silico and synthesized by GeneArt and presented in pMK vectors (Life Technologies, Thermo Fisher Scientific). The structure of the HDR template was designed following the previously published method (79). The full length of α- and β-chains of the TCR to be introduced, self-cleaving peptides P2 to ensure the separation of both TCR-chains, and a poly-A tail (bGHpA) were flanked by left and right homology arms (LHA and RHA). Both α- and β-chains consist of the human variable regions and the murine constant region with an additional disulfide bond (80), to facilitate the identification of re-expressed transgenic TCR with anti-mouse TCRβ antibody.

### Cas9 RNP production

CRISPR-Cas9 sgRNA (Integrated DNA Technologies) comprised of both crRNA and tracrRNA sequences were used. sgRNAs targeting both TRBC1 and TRBC2 (5’-GGAGAATGACGAGTGGACCC-3’) (81) and TRAC (5′-AGAGTCTCTCAGCTGGTACA-3′) (82) were mixed with Alt-R S.p.Cas9 Nuclease V3 (Integrated DNA Technologies) at 3:1 molar ratio and incubated for 15 min at room temperature.

### Orthotopic TCR replacement in primary human T cells

Frozen PBMCs were thawed and rested at 2-3x10^6^ cells/mL in TCM containing IL-2 (50 U/mL; BioLegend) and IL-15 (5 ng/mL; BioLegend) overnight at 37 °C, 5% CO_2_. PBMCs then were activated for 2 days with anti-human CD3/CD28 magnetic dynabeads (Thermo Fisher Scientific) at a beads to cells ratio of 1:1 in IL-2 (200 U/mL; BioLegend) and IL-15 (5 ng/mL; BioLegend) supplemented TCM. Activated PBMC cells (5-10 x10^6^) were harvested and electroporated with RNP mixture and HDR DNA templates (2.5 µg) using P3 Primary Cell 4D-Nucleofector™ X Kit S (Lonza) and a 4D Nucleofector X unit (Lonza) using EO115 electroporation program following the manufacturer’s protocol. Electroporated cells were seeded into a 24 well plate at a density of 5-10x10^6^ cells/mL in TCM containing IL-2 (200 U/mL; BioLegend). Mouse TCRβ-expressing T cells were sorted 3-5 days after electroporation and expanded with mixed lymphocyte reaction. The expanded TCR-transgenic T cells were subject to subsequent functional assays. When co-culturing with K562-EV/CD1a or GAS-infected K562-EV/CD1a cells for 4 hrs, expanded TCR-transgenic T cells were supplied with IL-6 (5 ng/mL; BioLegend), TNF-α (5 ng/mL; BioLegend), IL-2 (25 U/mL; BioLegend) and anti-CD3 (OKT3; 5 ng/ml; BioLegend) or IL-12 (1 ng/mL; BioLegend), IL-18 (1 ng/mL; BioLegend) and IL-2 (25 U/mL; BioLegend) to support CD1a-dependent cytokine production.

### Mice

Mice were bred and maintained under specific pathogen-free conditions at the University of Oxford, and all experiments were conducted in accordance with the approval of the UK Home Office. CD1a transgenic C57BL/6 mice (CD1a-Tg) were generated in Oxford, and have been previously described (68), and age-matched wild-type (WT) littermates were used as controls. Mice 6–10 weeks of age, males and females were used for experiments, and randomized into the different conditions.

### Skin challenge model

WT and CD1a-Tg mice were anesthetized by isoflurane inhalation and were treated intradermally with either 10 μl PBS or GAS (2x10^6^) in PBS to the dorsal side of the ear pinnae. Alternatively, WT and CD1a-Tg mice were anesthetized by isoflurane inhalation and were treated subcutaneously with either 20 μl PBS or GAS (4x10^6^) in PBS to the shaved back. Fourteen days after initial GAS challenges, 25 mg Aldara cream containing 5% imiquimod was applied to the dorsal and ventral sides of the ear pinnae daily for 6 days. Ear thickness was measured before and after challenges using a micrometer on indicated days, and photos of the challenge sites were documented. Mice were sacrificed, and ears, draining lymph nodes, spleen and blood were harvest for flow cytometry, histological and cytokine/chemokine profile analyses.

### Mouse tissue processing

Ears were dissected into small pieces and digested in 500 μl RPMI containing 10% FCS and 1 mg/ml Collagenase P (Roche Diagnostic) for an initial 1 hr at 37 °C. The digested tissues were briefly spin at 2,000 rpm for 5 min and the 200 μl clear supernatant was collected and stored at -20 °C for LEGENDplex analysis. The remaining tissue pellets were resuspended thoroughly with another 1 ml of RPMI + Collagenase P with another 1 hr incubation at 37 °C, and 100 µg/ml DNase I (Roche Diagnostic) was added for the final 30 min. The digested samples were passed through 70-μm cell strainers (BD Biosciences) and the digestion was stopped with 500 µl cold 10 mM EDTA in PBS, and a single cell suspension was obtained in FACS cell staining buffer (BioLegend). Auricular lymph nodes were harvested and meshed through 70-μm cell strainers (BD Biosciences) to obtain single cell suspensions in FACS cell staining buffer (BioLegend).

### Statistical analysis

Data are presented as mean ± standard error (SEM). Some results were calculated as the fold change of each condition to indicated control. Two-tailed paired/unpaired t test, one and two-way ANOVA tests were performed using GraphPad Prism version 9.00 (GraphPad Software).

## Supplementary Material

Data file S1

Supplementary material

## Data Availability

Single-cell RNA-seq files are available in the Gene Expression Omnibus under accession no. GSE206326. The code used in this manuscript for single-cell RNA-seq analysis is available in Zenodo project repository (). All data needed to evaluate the conclusions in the paper are present in the paper or the Supplementary Materials.

## One-sentence summary

GAS drives CD1a-autoreactive T cells and psoriatic inflammation
