# Supplementary material for: Group A streptococcus induces CD1a-autoreactive T cells and promotes psoriatic inflammation
Source: Sci Immunol. Author manuscript; Available in PMC 2024 Feb 23. (PMC7615662; doi:10.1126/sciimmunol.add9232)
Supplement: Supplementary material [file EMS194149-supplement-Supplementary_material.pdf]

Science Immunology
AAAS

Supplementary Materials for

Group A *Streptococcus* induces CD1a-autoreactive T cells and promotes psoriatic inflammation

Yi-Ling Chen *et al.*

Corresponding author: Graham S. Ogg, graham.ogg@ndm.ox.ac.uk

Sci. Immunol. **8**, eadd9232 (2023)
DOI: 10.1126/sciimmunol.add9232

The PDF file includes:

Supplementary Materials and Methods
Figs. S1 to S10
Table S1 and S2

Other Supplementary Material for this manuscript includes the following:

Data file S1
MDAR Reproducibility Checklist

Supplementary Materials

Supplementary materials and methods

Flow cytometry and antibodies.

Surface staining was performed at 4°C in FACS staining buffer (BioLegend) for 30 min. Subsequently, cells were washed and fixed with PBS solution contains 4% paraformaldehyde (BioLegend) in the dark for 10 minutes at room temperature. The data were acquired using LSRFortessa X-50 flow cytometer (BD Biosciences) and further analyzed with FlowJo (FlowJo LLC) software.

The following anti-human antibodies were used: Brilliant Violet 650-anti-CD3 (OKT3; BioLegend), Brilliant Violet 785-anti-CD3 (OKT3; BioLegend), BUV661-anti-CD3 (UCHT1; BD Biosciences), BUV805-anti-CD4 (SK3; BD Biosciences), PerCP/Cy5.5-anti-CD8 (SK1; BioLegend), PE/Cyanine7 anti-human TCR α/β (IP26; BioLegend), Brilliant Violet 711-anti-CD137 (4B4-1; BioLegend), Brilliant Violet 605 anti-CD45RA (HI100; BioLegend), BUV395 anti-CD45RO (UCHL1; BD Biosciences), Brilliant Violet 785 anti-CD25 (BC96; BioLegend), APC/Cy7 anti-CD69 (FN50; BioLegend), Alexa Fluor 700 anti-CD154 (24-31; BioLegend), FITC anti-human/mouse Cutaneous Lymphocyte Antigen (CLA) (HECA-452; BioLegend), PE anti-human TCR α/β (IP26; BioLegend), APC anti-human TCR α/β Antibody (IP26; BioLegend), Alexa Fluor 647 anti-human IFN-γ (4S.B3; BioLegend), and PE/Dazzle 594 anti-human GM-CSF (BVD2-21C11; BioLegend), PE anti-human IL-17A (BL168; BioLegend), and PE anti-human IL-22 (22URTI; eBioscience).

The following anti-mouse antibodies were used: BUV496 Rat Anti-Mouse CD4 (GK1.5; BD Biosciences), BUV395 Rat Anti-Mouse CD8a (53-6.7; BD Biosciences), BUV805 Rat Anti-Mouse CD45 (30-F11; BD Biosciences), Pacific Blue anti-mouse CD11a/CD18 (H155-78; BioLegend), Alexa Fluor 647 anti-mouse IL-22 (Poly5164; BioLegend), PE anti-mouse IFN-gamma (XMG1.2; BioLegend), PE anti-mouse IL-17A Antibody (TC11-18H10.1; BioLegend), PE/Cyanine7 anti-mouse F4/80 (BM8; BioLegend), Alexa Fluor 488 anti-mouse Ly-6C (HK1.4; BioLegend), Brilliant Violet 785 anti-mouse I-A/I-E (M5/114.15.2; BioLegend), Alexa Fluor 700 anti-mouse Ly-6G/Ly-6C (Gr-1) (RB6-8C5; BioLegend), Alexa Fluor 647 anti-mouse CD170 (Siglec-F) (S17007L; BioLegend), Brilliant Violet 650 anti-mouse CD11c (N418; BioLegend), PerCP/Cyanine5.5 anti-mouse/human CD11b (M1/70; BioLegend), PE anti-mouse/human CD207 (Langerin) (4C7; BioLegend), APC/Fire 750 anti-mouse CD69 (H1.2F3; BioLegend), PE anti-mouse TCR β chain (H57-597; BioLegend), Brilliant Violet 421 anti-mouse TCR β chain (H57-597; BioLegend).

Monocyte-derived DCs and LC-like cells

Monocyte-derived DCs were generated using CD14-expressing cells isolated using Magnetic-activated cell sorting with CD14 MicroBeads (Miltenyi Biotec) and differentiated in R10 containing 100 ng/ml GM-CSF and 10 ng/ml IL-4 at $1x10^6$ cells/ml concentration in 24-well plates. Additional 1 ml of R10 containing 100 ng/ml GM-CSF and 10 ng/ml IL-4 were supplied at day 3 for another 2-3 days. LC-like cells were generated by culturing $CD14^+$ monocytes in R10 containing 100 ng/ml GM-CSF, 10 ng/ml IL-4 and 10 ng/ml TGF-β. At day 3, cells were gently harvested, and the culture medium was removed and replaced with R10 containing 100 ng/ml GM-CSF and 10 ng/ml TGF-β and cultured for another 2-3 days.

ELISpot

ELISpot assay (Mabtech AB) was used to detect activation-induced IL-22 secretion from autologous polyclonal T cells upon coculture with GAS-infected Monocyte-derived DCs (mo-DCs) and LC-like cells. Mo-DCs and LC-like cells were infected with GAS (MOI= 5) overnight and the extracellular bacteria were removed before coculturing with T cells. The MultiScreen-IP sterile filter plates with hydrophobic poly (vinylidene fluoride) (Millipore) were coated with capture antibodies at 4°C overnight. *In vitro* cultured/infected mo-DCs or LC-like cells (2.5×10^4) were co-cultured with polyclonal T cells (5×10^4) per well with or without anti-HLA-A,B,C (10 µg/ml, W6/32; BioLegend) and HLA-DR (10 µg/ml, L243; BioLegend) antibodies and incubated at 37°C and 5% CO_2 overnight. In some wells, mo-DCs and LC-like cells were pretreated with anti-CD1a blocking antibody (10 µg/ml) or IgG1 isotype control (10 µg/ml; BioLegend) for 1 hr before the addition of T cells. PMA (5 ng/ml) and ionomycin (5 ng/ml) stimulation or T cells alone were set up as positive controls or negative controls, respectively. After incubation, plates were washed and incubated with biotin-linked anti–IL-22 monoclonal antibody for 2 hrr. After washing, the plates were incubated for 2 hr with streptavidin–alkaline phosphatase and were developed using the alkaline phosphatase conjugate substrate kit (Bio-Rad). The numbers of IL-22-producing T cells were counted using the automated ELISpot reader (ELISpot Reader Classic; AID Autoimmun Diagnostika GmbH).

Pan-T cell stimulation

T cells were treated with phorbol 12-myristate 13-acetate (PMA) and ionomycin (Cell Stimulation Cocktail (500X); eBioscience) and Brefeldin A (BioLegend) for 4 hrs. Cells were assessed by intracellular cytokine staining of cytokine production (IL-22 or IL-17A) using Inside Stain Kit (Miltenyi Biotec) following the manufacturer's instructions and stained with antibodies directed against cytokines. Data were acquired using LSRFortessa X-50 flow cytometer (BD Biosciences) and further analyzed with FlowJo (FlowJo LLC) software.

scRNA-seq data analysis

Seurat (v4.1.0, R package (*78*)) was used to perform data normalisation, scaling, transformation, clustering, dimensionality reduction, differential expression analysis and most of the data visualisation. Clustering analysis was performed by applying weighted nearest neighbors (WNNs) analysis in Seurat v4 to measure and integrate multimodal single-cell sequencing data (*79*). Using first 30 PCs from RNA and first 20 PCs from protein, closest multimodal neighbours were calculated based on a weighted combination of RNA and protein similarities. Cells were then clustered using Smart local Moving (SLM) algorithm for modularity optimization with WNN graph as input and the resolution parameter was set to 0.6. Cell cluster of data based on a weighted combination of RNA and protein data was visualised with uniform manifold approximation and projection (UMAP) algorithm and phenotypically distinct clusters were identified. The differential expression gene (DEG) analysis among indicated groups were performed using "FindMarkers" functions with "bimod" test method and 0.25 logfc.threshold. Genes with adjusted p value < 0.05 was used. T cell annotations were determined based on feature expression of surface markers CD4, CD8, CD45RA, CD45RO, CCR7, CD25 and CD11a. In short, T subsets were broadly classified as naïve T cells ($CD45RA^{+}$CD45RO-$CCR7^{+}$), and antigen-experienced T cells (remaining subsets).

Pseudotime ordering

Single-cell trajectories were constructed using the Monocle 3 R package (*51, 80, 81*); small irrelevant clusters with less than 165 cells (clusters 13, 14, and 15) were removed and the remaining T cells were placed on the pseudotime trajectories based on changes in the transcriptomes, with unstimulated T cells assigned as the root node for ordering. This was initially done with combined $CD4^{+}$ and $CD8^{+}$ populations. The pseudotime information was incorporated into the Seurat object and 3769 DEGs were found using "FindAllMarkers" functions along the pseudotime bins with "wilcox" test method and 0.25 logfc.threshold. Genes with adjusted p value < 0.05 were used.

TCR motif analysis

Single-cell TCR clonotypes were assembled using Cellranger VDJ software, Immunarch v0.8.0 (*82*) and scRepertoire v1.7.2 (*83*) R packages were used to incorporate and compute TCR repertoire information for individual samples. Five hyperexpanded clones (≥70 cells) were removed and expanded T cells with unique clonotype (at least ≥ 2 cells per clonotype) were separated based on their positions on their pseudotime trajectories into four groups: 175 $CD4^{+}$ early, 281 $CD4^{+}$ late, 423 $CD8^{+}$ early, and 226 $CD8^{+}$ late population, with 62, 97, 99, and 73 unique TCR clonotypes distributed across all donors, respectively. The CDR3 sequences of these TCR clonotypes were subjected to GLIPH2 analysis and top motif candidates were returned (*52*). The proportion of TCR clonotypes containing each motif was calculated among each group.

Bead-based immunoassays

For evaluating the levels of the cytokines in the co-culture supernatant, mice skin extracts and plasma, a cytometric bead array was perform using the following LEGENDplex™ kits (BioLegend) following the manufacturer's guidelines: Human CD8 Panel (13-plex), MU Macrophage/Microglia Panel (13-plex), MU Inflam Panel (13-plex) and MU Macrophage/Microglia Panel (13-plex). The data were acquired using LSRFortessa X-50 flow cytometer (BD Biosciences) and the analysis was performed by using LEGENDplex™ Data Analysis Software.

CD1a lipid loading and isoelectric focusing assay (IEF)

CD1a (10 μg) was treated with a 100X molar excess of LPC 18:1 or LPC 18:0 (Avanti Polar Lipids) in Tris Buffer saline containing 0.25% CHAPS or vehicle alone (mock) for 16 h at 37 °C. Loaded CD1a was then separated on an isoelectric focusing (IEF) pH 3-7 gel (Novex) by running for 1 h at 100 V, 1 h and 200 V and 30 min at 500 V. The gel was then fixed with 12% TCA for 15 min and stained with heated SimplyBlue SafeStain (Invitrogen) for 7 min, and destained in DI water overnight.

TCR sequencing

Single T cells were sequenced essentially as described previously(*84*). Briefly, cDNA was synthesized from single-cell RNA templates using a Superscript Vilo cDNA Synthesis Kit (Thermo Fisher Scientific), and products were amplified separately using a multiplex PCR with nested primers for TRA or TRB. Amplicons were visualized on a 2% agarose gel and purified using a Nucleospin Gel and PCR Clean Up Kit (Macherey-Nagel). TCR sequencing was performed using reverse primers for TRAC or TRBC (Genewiz). These TCR sequences were further validated with previously described methods (*85*) using RNA extracted from individual clones with a RNeasy Plus Micro kit (Qiagen), according to the manufacturer's instructions. cDNA was generated by template-switch reverse transcription with SMARTScribe Reverse Transcriptase (Clontech) and a template-switch oligo (TSO: 5′-AAGCAGTGGTATCAACGCAGAGTGGCCATTACGGCCrGrGrG-3′) and primers designed for the Trac and Trbc genes (TraRv0, 5′-TCA GCT GGA CCA CAG CCG CAG-3′; TrbRv0, 5′-CAG TAT CTG GAG TCA TTG A-3′). Two rounds of nested PCR(First PCR_FwD primer, 5′-CAC GAC GCT CTT CCG ATC TGTCAACTC CAG TGG TAT CAA CGC AGA GTA C-3′; first PCRTraRv, 5′-TAC ACG GCA GGG TCA GGG T-3′; first PCRTrbRv, 5′-TGC TTC TGA TGG CTC AAA CAC-3′; second PCR FwD primer, 5′-CAC TCT TTC CCT ACA CGA CGC TCT TCC GAT C-3′; second PCRTraRv, 5′-TGG AGT TCA GAC GTG TGC TCT TCC GAT CTG GGT CAG GGT TCT GGA TAT-3′; second PCRTrbRv, 5′-TGG AGT TCA GAC GTG TGC TCT TCC GAT CTA CAC STT KTT CAG GTC CTC-3′) were performed to amplify TCR fragment with Phusion High-Fidelity PCR Master Mix (New England BioLabs) and the TCR amplicons were sequenced using a 48 capillary ABI-3730 DNA analyser (Thermo Fisher Scientific). Post analysis was performed with SnapGene (Dotmatics).

CD1a bead-bound assay

Biotinylated CD1a (4 ug, produced in house) was treated with a 100X molar excess of LPC 18:1 or LPC 18:0 (Avanti Polar Lipids) or vehicle alone (mock) and coated onto the magnetic M-450 Epoxy Dynabeads ($12x10^6$) together with anti-CD3 (Clone OKT3), anti-CD28 (Clone 15E8), and anti-CD2 (Clone LT2) (150 ng each, Miltenyi Biotec) overnight with rotation at room temperature. The unbound CD1a and antibodies were removed by washing beads with culture medium three times on a magnet. Beads ($5x10^5$) were co-cultured with engineered T cells ($1\text{-}5x10^5$) for 4 hrs supplied with IL-12 (1 ng/mL; BioLegend), IL-18 (1 ng/mL; BioLegend), IL-2 (25 U/mL; BioLegend), and 2.5 μg/ml anti-CD11a (HI111; BioLegend) to support CD1a-dependent cytokine production. Brefeldin A (BioLegend) was added, and activation of T cells were assessed by intracellular cytokine staining of cytokine production (IFNγ or GM-CSF) using an Inside Stain Kit (Miltenyi Biotec) following manufacturer's instructions and stained with antibodies against cytokines.

Intracellular staining and cytokine secretion assay of mouse T cells

Cells were stained with antibodies against surface antigens in FACS cell staining buffer (BioLegend) at 4 °C for 30 min. Zombie Fixable Viability dyes (BioLegend) were used to exclude dead cells. For intracellular cytokine staining, *ex vivo* isolated cells were stained with surface marker first, and cells were fixed and permeabilized with *FOXP3* Fix/Perm Buffer (BioLegend) or Inside Stain Kit (Miltenyi Biotec) following manufacturer's instructions and stained with antibodies against cytokines. For murine *in vitro* CD1a cytokine secretion assay, biotinylated CD1a (4 ug, produced in house) was coated onto the magnetic M-450 Epoxy Dynabeads ($12x10^6$) together with anti-mouse CD3ε (clone 145-2C11, 150 ng; Miltenyi Biotec) and anti-mouse CD28 (Clone 37.51, 150 ng; Miltenyi Biotec) overnight with rotation at room temperature. The unbound CD1a and antibodies were removed by washing beads with R10 three times on a magnet. Beads ($5x10^5$) were co-cultured with mouse T cells ($1\text{-}5x10^5$) isolated from lymph nodes or spleens for 4 hrs in R10 at 37 °C, and assessed their activation using mouse IFNγ Cytokine Secretion Assays (Miltenyi Biotec) following the manufacturer's instructions. The data were acquired using LSRFortessa X-50 flow cytometer (BD Biosciences) and further analyzed with FlowJo (FlowJo LLC) software.

Histology and Confocal imaging

Murine ear skin was snap frozen in OCT Embedding Matrix (Scigen) and stored at −80°C until cutting. Cryosections (6μm) were cut using a Leica cryostat to attach onto Superfrost Plus slides and allow to air-dry for 30 min and stored at −80°C. For histology imaging, slides were stained with Hematoxylin and Eosin Stain Kit (Vector Laboratories Ltd) following the manufacturer's protocol. Images were taken using a Revolve Microscope (Echo). For fluorescent imaging, tissues were rehydrated with PBS for 10 min and 0.15% hydrogen peroxide solution was applied for 5 min to quench the endogenous peroxidase activity. Avidin/Biotin Blocking Kit (Vector Laboratories Ltd) and 10% goat serum

(Thermo Fisher Scientific) was used to block endogenous biotin and nonspecific binding of antibodies, respectively. Anti-CD1a antibody (OKT6; 1:100; produced in-house and conjugated with Biotin using EZ-Link™ NHS-Biotin (Thermo Fisher Scientific)) and FITC Rabbit polyclonal to Streptococcus group A (1:10; Abcam) were diluted in 1%BSA/5% Goat Serum and incubated overnight at 4°C. Alexa Fluor 594 Tyramide SuperBoost kit (streptavidin; Thermo Fisher Scientific) was used to enhance signals: After washing away the primary antibodies, HRP-conjugated streptavidin was added to the tissues and incubated at 4°C overnight. After removing excess streptavidin-HRP, tyramide working solution was added to the tissues for 8 min at room temperature, and the reaction was stopped with Reaction Stop Reagent. Slides were mounted with ProLong Diamond Antifade Mount (Thermo Fisher Scientific) and kept at 4°C in dark. Images were obtained using confocal microscopy (Zeiss LSM 780 Confocal Microscope-Inverted Microscope; 25×/0.8 Imm Korr DIC M27; Zen software), and Fiji was used for image processing.

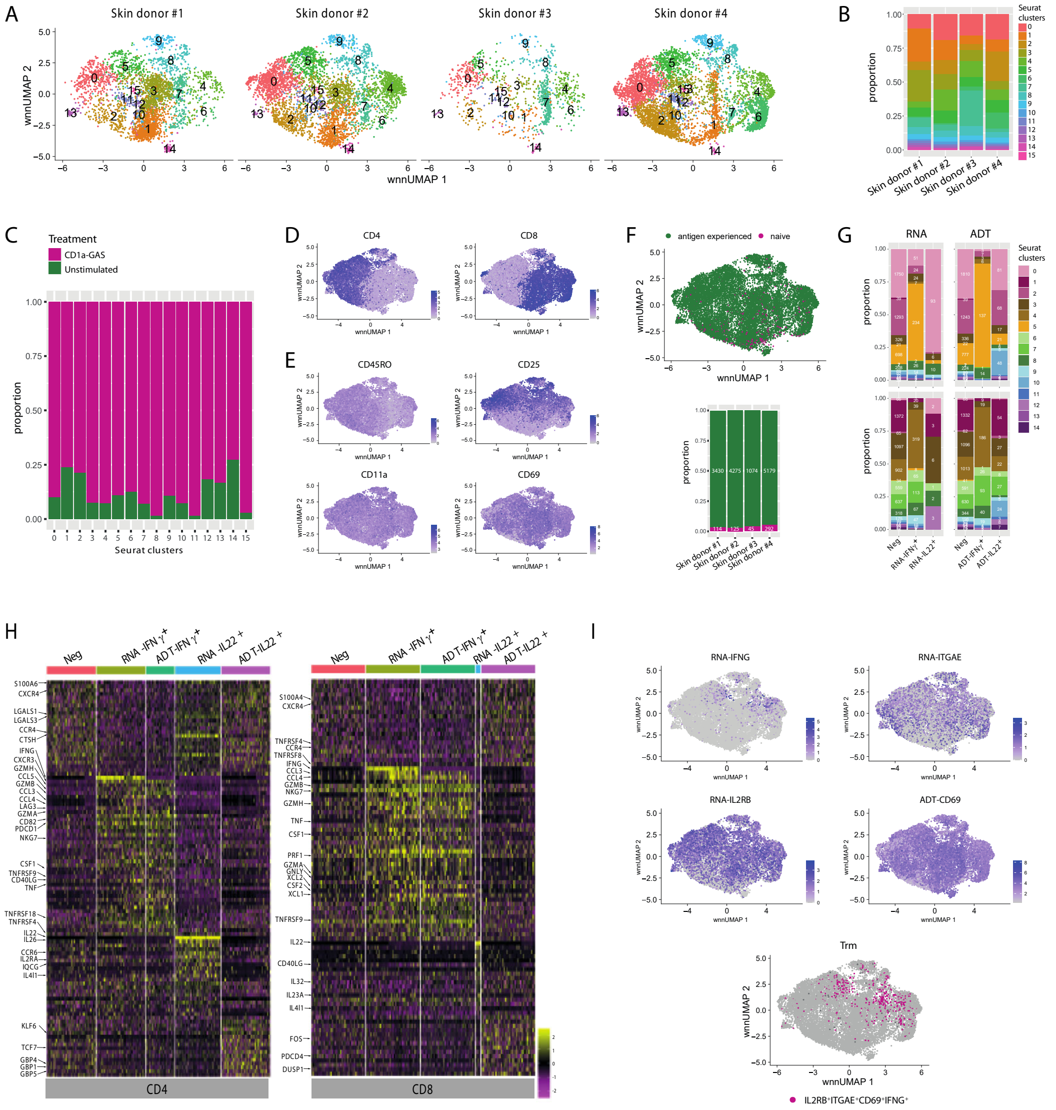

Figure S1. Single-cell heterogeneity of skin CD3$^+$ cells after co-culture with GAS-infected K562-CD1a cells. **(A)** UMAP plot showing unbiased clustering of the skin CD3$^+$ cells across four donors. **(B)** Bar chart showing the cluster distribution of T cells derived from each donor. **(C)** Bar chart showing the distribution of T cells derived from each treatment condition. **(D)** Feature plot showing the expression of CD4 and CD8 protein in skin CD3$^+$ cells. **(E)** Feature plot showing the expression of CD45RO, CD25, CD11a and CD69 protein in skin CD3$^+$ cells and **(F)** UMAP plot (top panel) and bar chart (bottom panel) colored by antigen experience of T cells. **(G)** Proportion and cell numbers of the ADT-IL-22$^+$, RNA-IL-22$^+$, ADT-IFNγ$^+$, RNA-IFNγ$^+$ and IL22-IFNγ- (Neg) skin T cells within each Seurat clusters**.** **(H)** Heat map depicting selective top 30 genes differentially expressed in the five subgroups of CD4$^+$ or CD8$^+$ T cells. **(I)** Feature plot showing the gene expression of IFNG, ITGAE, IL2RB and protein level of CD69 in skin CD3$^+$ cells, and activated tissue-resident memory cells were defined as IL2RB$^+$ITGAE$^+$CD69$^+$ showing in magenta embedded in the UMAP.

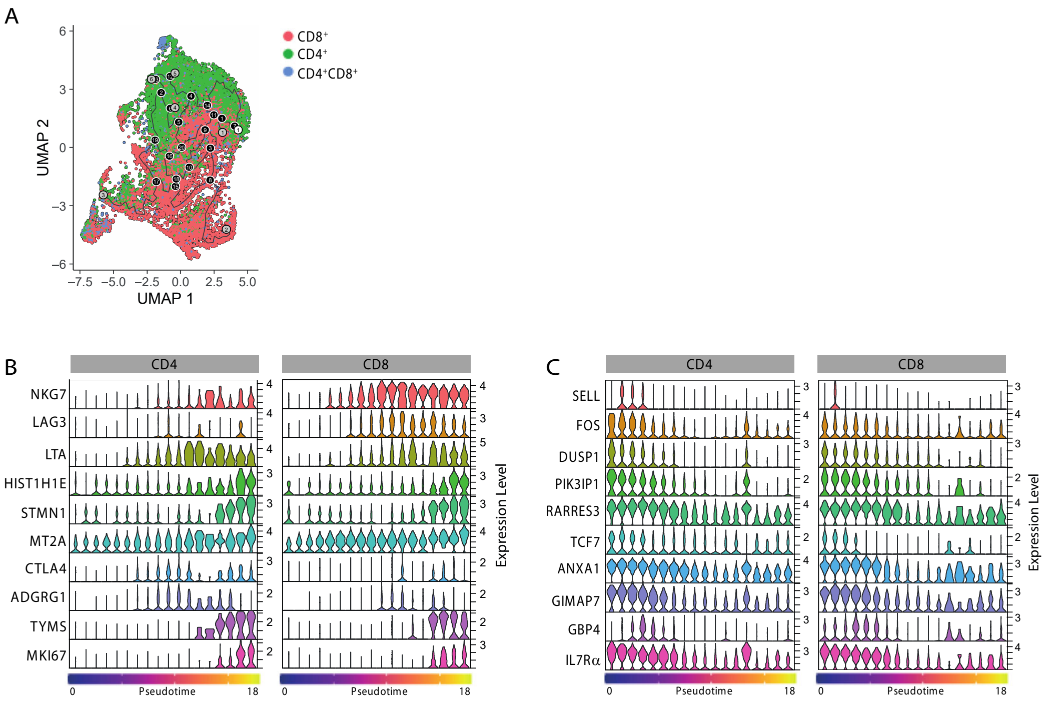

Figure S2. Pseudotime trajectory analysis of skin CD3^{+} cells after co-culture with GAS-infected K562-CD1a cells shows acquisition of broad functional potential. **(A)** Trajectory visualization with cells ordered and colored according to their CD4 and CD8 expression. Violin plots show expression patterns of genes positively **(B)** and negatively **(C)** changed in skin CD4^{+} and CD8^{+} T cells over pseudotime trajectory in response to CD1a-GAS presentation. (genes with fold change ≥ 0.5, adjusted $p < 0.05$)

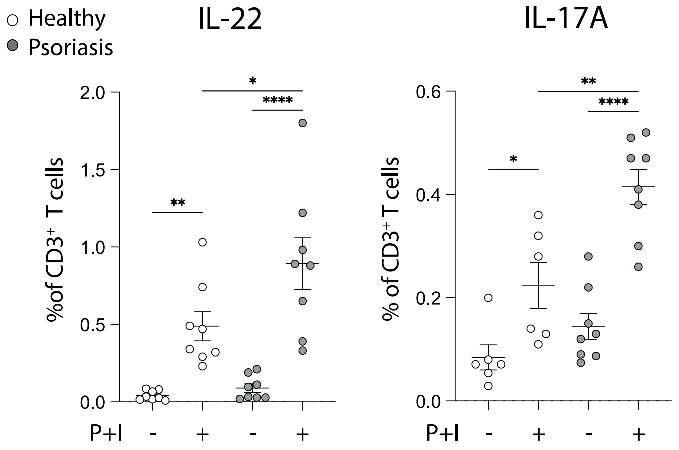

Figure S3. Psoriatics have elevated frequencies of IL-22 and IL-17A-producing blood T cells after pan-T cell stimulation.

Blood T cells with the ability to produce IL-22 (A) and IL-17A (B) after treating with Pan-T cell stimuli (PMA and ionomycin; P+I) and analyzed by intracellular flow cytometry (n=8). Each symbol represents an individual donor (mean ± SEM). *P < 0.05, **P < 0.01 and ****P < 0.0001; two-way ANOVA with Tukey's post hoc test.

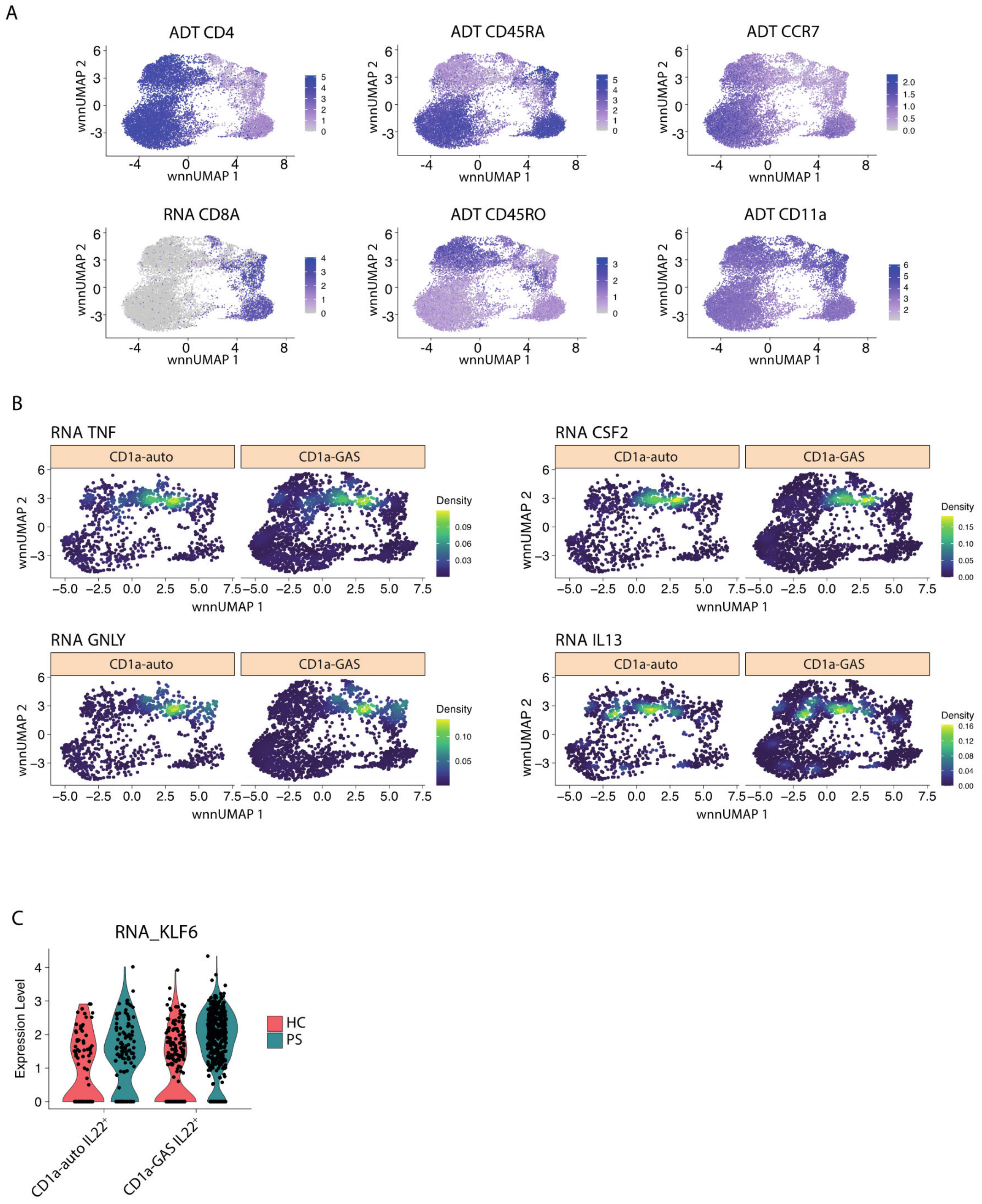

Figure S4. Single-cell heterogeneity of blood CD3$^+$ cells after co-culture with GAS-infected K562-CD1a cells. **(A)** Feature plot showing the expression of CD4, CD45RA, CCR7, CD45RO and CD11a protein, and CD8A mRNA expression in blood CD3$^+$ cells. **(B)** Nebulosa plots showing gene expression density of *TNF*, *CSF2*, *GNLY*, and *IL13* from sequenced blood CD3$^+$ cells. **(C)** Violin plot showing expression level of the *KLF6* in IL-22-producing blood CD4$^+$ T cells isolated from healthy and psoriasis patients.

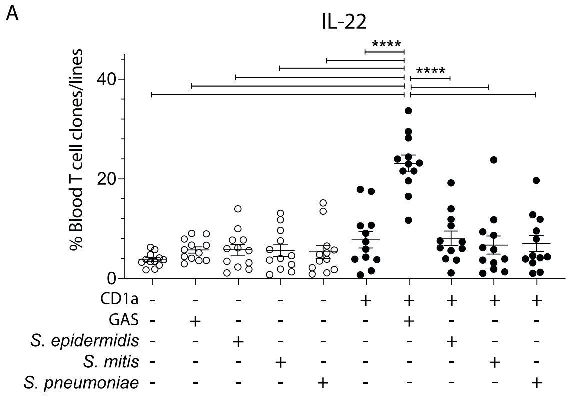

Figure S5. Blood and skin CD1a-reactive T cell lines show increased proportional responsiveness to GAS compared to other streptococcal and staphylococcal species tested. Production of IL-22 from expanded CD1a-reactive T cell clones/lines from blood **(A)** or skin **(B)** detected by Secretion assay after 4-hour co-culturing with control, GAS-, *S. epidermidis*-, *S. mitis*-, and *S. pneumoniae*-infected K562 cells (MOI=50) (n=6-12). Each symbol represents an individual T cell clone/line (mean $\pm$ SEM). ****$P < 0.0001$; two-way ANOVA with Tukey's post hoc test. Data are representative of more than three independent experiments.

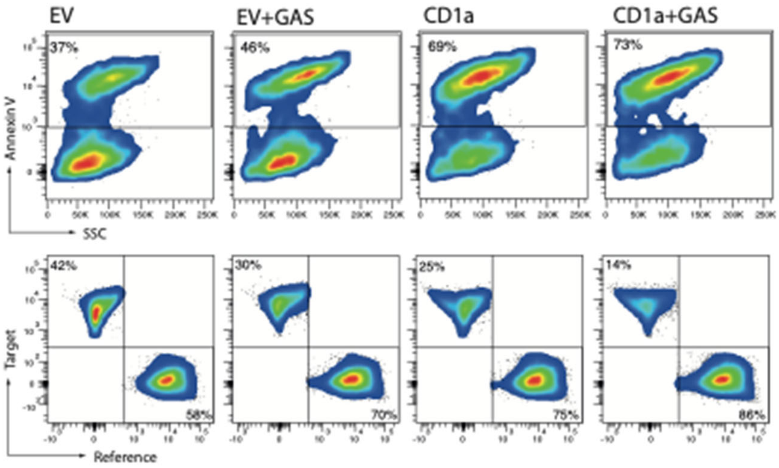

Figure S6. Gating strategy of the apoptotic cells (Annexin V$^+$) and the proportion of target and reference cells. Representative flow cytometry images of the percentage of Annexin V$^+$ cells, and the proportion of target and reference cells after co-culture of CD1a-reactive CD8$^+$ T cells with GAS-infected or uninfected K562-CD1a cells.

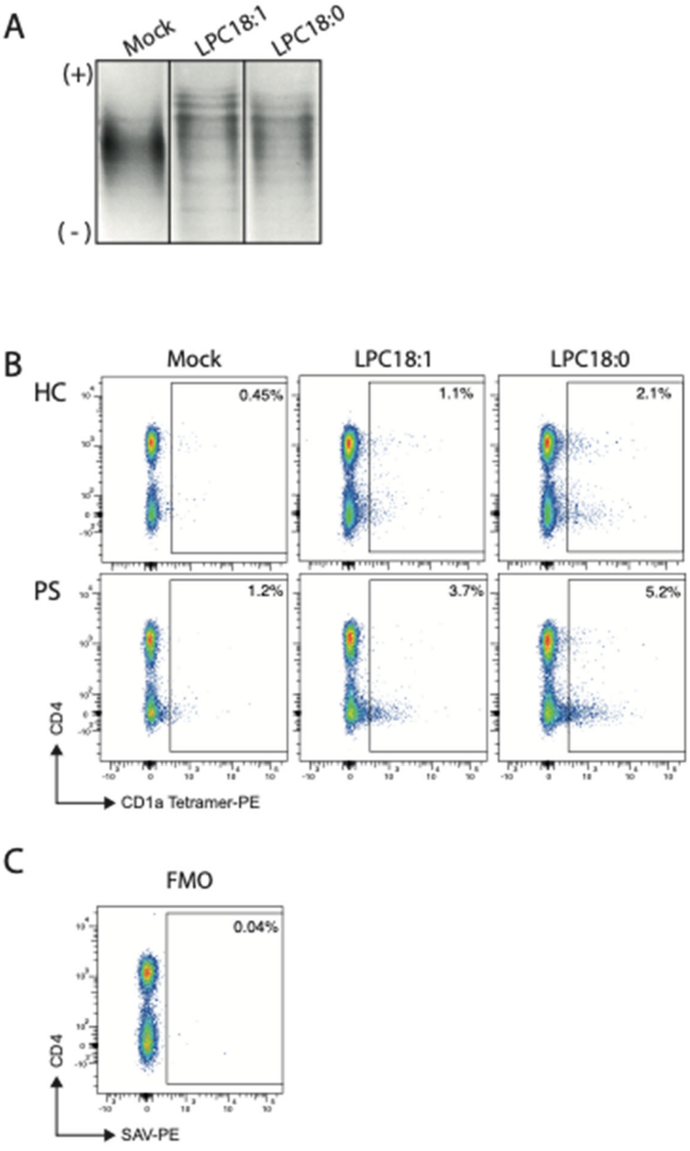

Figure S7. Detection of circulating CD1a-tetramer-binding T cells. **(A)** Isoelectric point dependent migration of mock and LPC-treated CD1a protein. Mock: vehicle control TBS 0.25% CHAPS. Data shown representative of two independent experiments. **(B)** Representative flow cytometry images depicting CD1a tetramer staining of $CD3^+$ T cells from healthy control and PS patients. **(C)** Representative flow cytometry image of negative control staining with streptavidin-PE (SAV-PE)

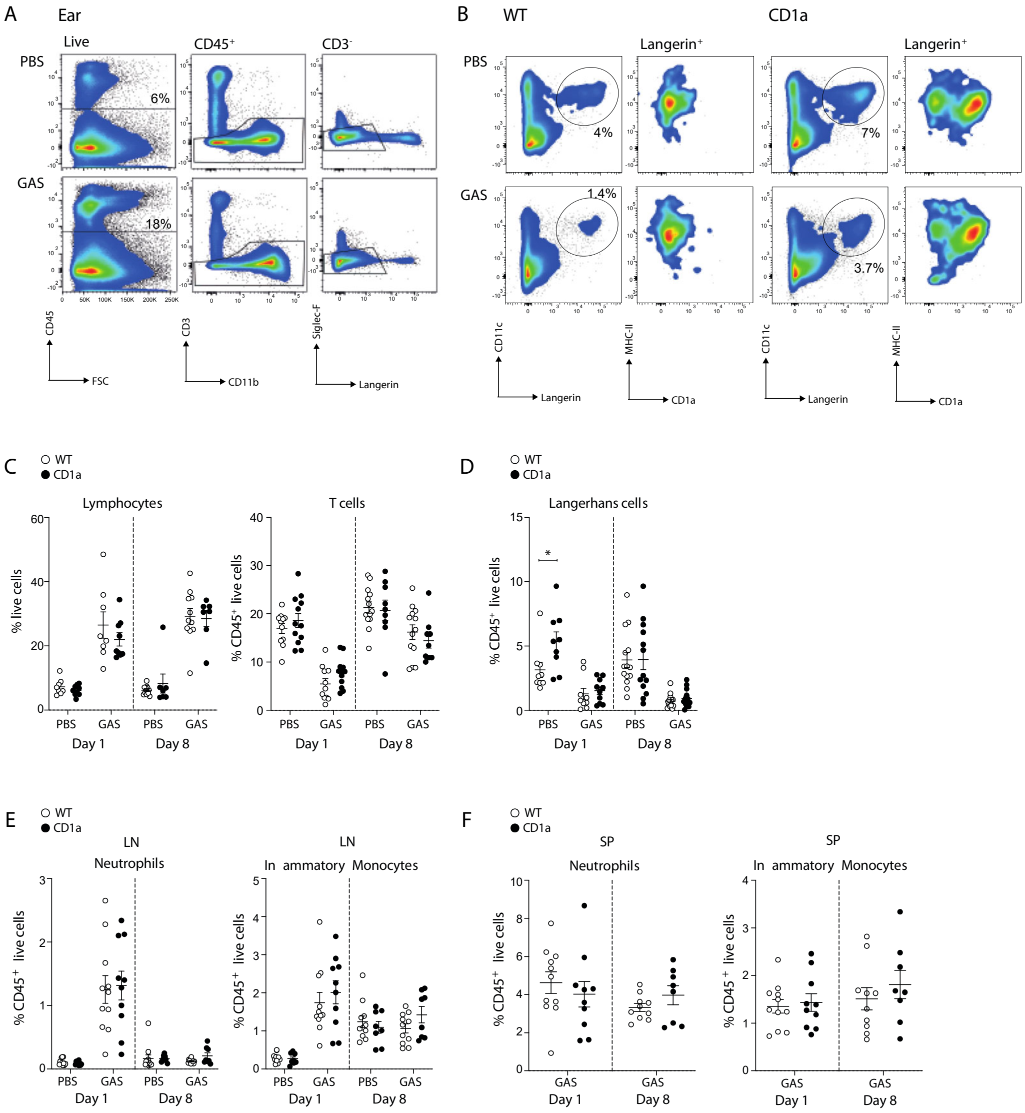

Figure S8. Composition of skin infiltrating lymphoid and myeloid cells in GAS-infected mice. **(A-B)** Representative skin flow cytometry plots showing the gating strategy of CD45$^+$ skin cells and Langerhans cells (CD45$^+$CD3$^-$Siglec-F$^-$CD11c$^+$Langerin$^+$) with MHC-II and CD1a expression levels. Percentages of skin infiltrating lymphocytes **(C,** left panel**)**, T cells **(C**, right panel**)**, Langerhans cells **(D)**, LN-accumulating neutrophils and inflammatory monocytes **(E)**, and spleen-accumulating neutrophils and inflammatory monocytes **(F)** from mice infected with GAS after flow cytometry analysis (n=8-14). Each symbol represents an individual mouse (mean $\pm$ SEM). *P < 0.05; two-way ANOVA with Šídák's post hoc test (C, D, E), or two-tailed unpaired t test (F). Data are representative of more than three independent experiments.

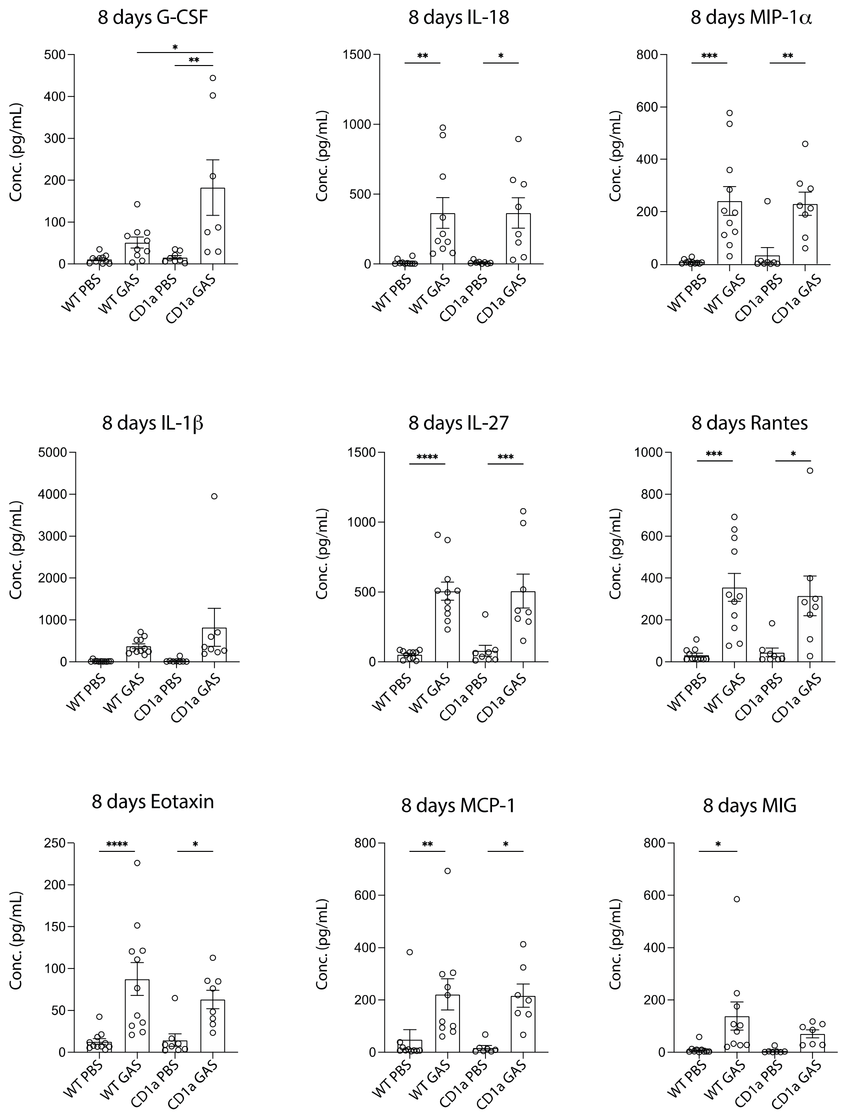

Figure S9. Cytokine profiles of ear skin extracts of PBS- and GAS-infected WT and CD1a transgenic mice. Cytokine concentrations in ear skin extracts of GAS-infected WT and CD1a transgenic mice were analyzed by bead-based immunoassays after 1-day and 8-day GAS inoculation (n=7-14). Each symbol represents an individual mouse (mean + SEM). $^{*}P < 0.05$, $^{**}P < 0.01$, $^{***}P < 0.001$ and $^{****}P < 0.0001$; two-way ANOVA with Tukey's post hoc test. Data are representative of more than three independent experiments.

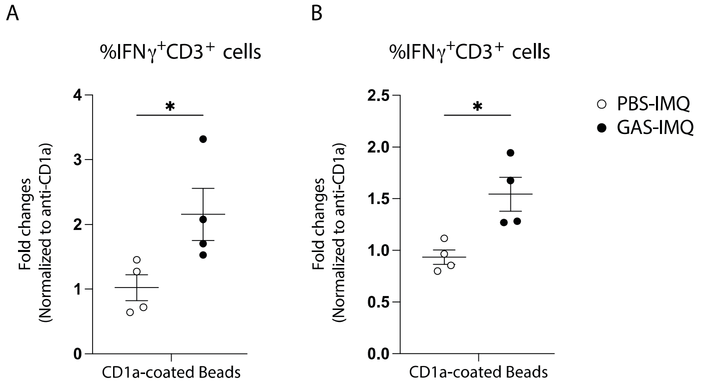

Figure S10. CD1a-reactivity of hCD1a-transgenic murine T cells *in vitro*. Percentage of IFNγ-secreting $CD3^+$ cells from draining lymph node **(A)** and spleen **(B)** harvested from CD1a-Tg mice with or without prior GAS infection after *in vitro* stimulated with bead-bound CD1a (n=4). *P < 0.05; two-tailed unpaired T test. The data were graphed as the fold change of CD1a blocking condition. Data are representative of two independent experiments.

Supplementary table I: Psoriasis clinical metadata.

PASI, psoriasis area and severity index

Figure 5A	**N**	15
	**Age (mean ± SEM)**	48.27 ± 3.65
	**Sex (Female / Male)**	6 / 9
	**PASI (mean ± SEM)**	4.73 ± 0.9
Single-cell CITEseq	**N**	3
	**Age (mean ± SEM)**	34.67 ± 6.57
	**Sex (Female / Male)**	1 / 2
	**PASI (mean ± SEM)**	3.33 ± 1.2
Figure 6I	**N**	17
	**Age (mean ± SEM)**	53.77 ± 3.03
	**Sex (Female / Male)**	8 / 9
	**PASI (mean ± SEM)**	4.47 ± 0.79

Supplementary table II: TotalSeq-C antibody pool

	Description	Clone	Barcode
C0007	anti-human CD274 (B7-H1, PD-L1)	29E.2A3	GTTGTCCGACAATAC
C0008	anti-human CD273 (B7-DC, PD-L2)	24F.10C12	TCAACGCTTGGCTAG
C0046	anti-human CD8	SK1	GCGCAACTTGATGAT
C0047	anti-human CD56	5.1H11	TCCTTTCCTGATAGG
C0061	anti-human CD117 (c-kit)	104D2	AGACTAATAGCTGAC
C0063	anti-human CD45RA	HI100	TCAATCCTTCCGCTT
C0071	anti-human CD194 (CCR4)	L291H4	AGCTTACCTGCACGA
C0072	anti-human CD4	RPA-T4	TGTTCCCGCTCAACT
C0080	anti-human CD8a	RPA-T8	GCTGCGCTTTCCATT
C0085	anti-human CD25	BC96	TTTGTCCTGTACGCC
C0087	anti-human CD45RO	UCHL1	CTCCGAATCATGTTG
C0088	anti-human CD279	EH12.2H7	ACAGCGCCGTATTTA
C0089	anti-human TIGIT (VSTM3)	A15153G	TTGCTTACCGCCAGA
C0102	anti-human CD294 (CRTH2)	BM16	TGTTTACGAGAGCCC
C0140	anti-human CD183 (CXCR3)	G025H7	GCGATGGTAGATTAT
C0141	anti-human CD195 (CCR5)	J418F1	CCAAAGTAAGAGCCA
C0143	anti-human CD196 (CCR6)	G034E3	GATCCCTTTGTCACT
C0144	anti-human CD185 (CXCR5)	J252D4	AATTCAACCGTCGCC
C0145	anti-human CD103 (Integrin αE)	Ber-ACT8	GACCTCATTGTGAAT
C0146	anti-human CD69	FN50	GTCTCTTGGCTTAAA
C0147	anti-human CD62L	DREG-56	GTCCCTGCAACTTGA
C0148	anti-human CD197 (CCR7)	G043H7	AGTTCAGTCAACCGA
C0149	anti-human CD161	HP-3G10	GTACGCAGTCCTTCT
C0151	anti-human CD152 (CTLA-4)	BNI3	ATGGTTCACGTAATC
C0152	anti-human CD223 (LAG-3)	11C3C65	CATTTGTCTGCCGGT
C0153	anti-human KLRG1 (MAFA)	SA231A2	CTTATTTCCTGCCCT
C0154	anti-human CD27	O323	GCACTCCTGCATGTA
C0158	anti-human CD134 (OX40)	Ber-ACT35 (ACT35)	AACCCACCGTTGTTA
C0159	anti-human HLA-DR	L243	AATAGCGAGCAAGTA
C0160	anti-human CD1c	L161	GAGCTACTTCACTCG
CD169	anti-human CD366 (Tim-3)	F38-2E2	TGTCCTACCCAACTT
C0170	anti-human CD272 (BTLA)	MIH26	GTTATTGGACTAAGG
C0171	anti-human/mouse/rat CD278 (ICOS)	C398.4A	CGCGCACCCATTAAA

C0174	anti-human CD58 (LFA-3)	TS2/9	GTTCCTATGGACGAC
C0176	anti-human CD39	A1	TTACCTGGTATCCGT
C0185	anti-human CD11a	TS2/4	TATATCCTTGTGAGC
C0189	anti-human CD244 (2B4)	C1.7	TCGCTTGGATGGTAG
C0355	anti-human CD137 (4-1BB)	4B4-1	CAGTAAGTTCGGGAC
C0360	anti-human CD357 (GITR)	108-17	ACCTTTCGACACTCG
C0386	anti-human CD28	CD28.2	TGAGAACGACCCTAA
C0389	anti-human CD38	HIT2	TGTACCCGCTTGTGA
C0390	anti-human CD127 (IL-7Rα)	A019D5	GTGTGTTGTCCTATG
C0394	anti-human CD71	CY1G4	CCGTGTTCCTCATTA
C0402	anti-human CD1a	HI149	GATCGTGTTGTGTTA
C0436	anti-Biotin	1D4-C5	CGGTATATCAACAGA
C0576	anti-human CD49d	9F10	CCATTCAACTTCCGG
C0577	anti-human CD73 (Ecto-5'-nucleotidase)	AD2	CAGTTCCTCAGTTCG
C0804	anti-human CD186 (CXCR6)	K041E5	GACAGTCGATGCAAC
C0815	anti-human CCR10	6588-5	ATCTGTATGTCACAG
C0867	anti-human CD94	DX22	CTTTCCGGTCCTACA
C0896	anti-human CD85j (ILT2)	GHI/75	CCTTGTGAGGCTATG
C0901	anti-human GARP (LRRC32)	7B11	AGGTATGGTAGAGTA
C0911	anti-phycoerythrin (PE)	PE001	TGACCAGTTCCGCAT
C0987	anti-Allophycocyanin (APC)	APC003	TTAACCGTCTCCCTT
C1024	anti-human CD120b	3G7A02	GCGCAACTCCTTGTA
C1137	anti-human CD147	HIM6	CTTACGATTAAGAGC
